# The structure and function of membrane protein in coronavirus infection and its applications in the development of vaccines and therapeutic drugs

**DOI:** 10.3389/fmicb.2026.1762041

**Published:** 2026-02-18

**Authors:** Shengnan Jiang, Jianbo Yuan, Qingyang Li, Ziyan Song, Lijing Cao, Zhenhui Song, Xingcui Zhang

**Affiliations:** 1 College of Veterinary Medicine, Southwest University, Rongchang, Chongqing, China; 2 Immunology Research Center, Institute of Medical Research, Southwest University, Chongqing, China; 3 Rongchang Vocational Education Center, Chongqing, China

**Keywords:** coronavirus, glycosylation, membrane protein, therapeutic, vaccine

## Abstract

Coronaviruses have long posed significant harm to human and animal health, causing a variety of diseases. The membrane (M) protein of coronaviruses is one of the four major structural proteins and a key component of the viral structure, playing an important role in viral assembly, budding, and immunomodulation. In this paper, we systematically reviewe the structural and functional characteristics of the M protein, including its three transmembrane domains, N-terminal glycosylation and C-terminal oligomerization domain. In terms of function, we focus on the mechanistic roles of the M protein in viral envelope formation and the nucleocapsid packaging, as well as the newly discovered immune evasion strategy of regulating host innate immune signaling pathways. In addition, we also summarize the applications of M protein in preventing and controlling coronavirus infection and mitigating its adverse effects.

## 1 Introduction

Coronavirus (CoV) infection has become one of the most important infectious diseases affecting both humans and animals. Various CoVs have been isolated or detected in animals such as swine, bats, camels, deer, mink, canines and felines. With a broad host range encompassing avian and mammalian species, CoVs cause a variety of serious diseases that pose significant threats to the agricultural industry (Lin et al., 2021; Malik, 2020; Miranda et al., 2021). Coronaviruses belong to the order Nidovirales, family Coronaviridae, subfamily Orthocoronavirinae. Based on genomic sequence, serotype and other characteristics, members of this subfamily are divided into four genera, namely Alpha-coronavirus (α-CoV), Beta-coronavirus (β-CoV), Gamma-coronavirus (γ-CoV) and Delta-coronavirus (δ-CoV) (Fung and Liu, 2019; Malik, 2020). According to the current database of the International Committee on Taxonomy of Viruses (ICTV) taxonomy release, there are 26 species of α-CoVs, 15 species of β-CoVs., 5 species of γ-CoVs., and 7 species of δ-CoVs., Over the past 20 years, seven types of CoVs., that can infect humans and pose a threat to human health have been identified. Members of β-CoV genus are generally more harmful. Among them, Middle East Respiratory Syndrome Coronavirus (MERS-CoV), Severe Acute Respiratory Syndrome Coronavirus (SARS-CoV) and SARS-CoV-2 are highly pathogenic CoVs that can cause severe illness and even death; these viruses also possess a greater potential for widespread transmission.

**FIGURE 1:**
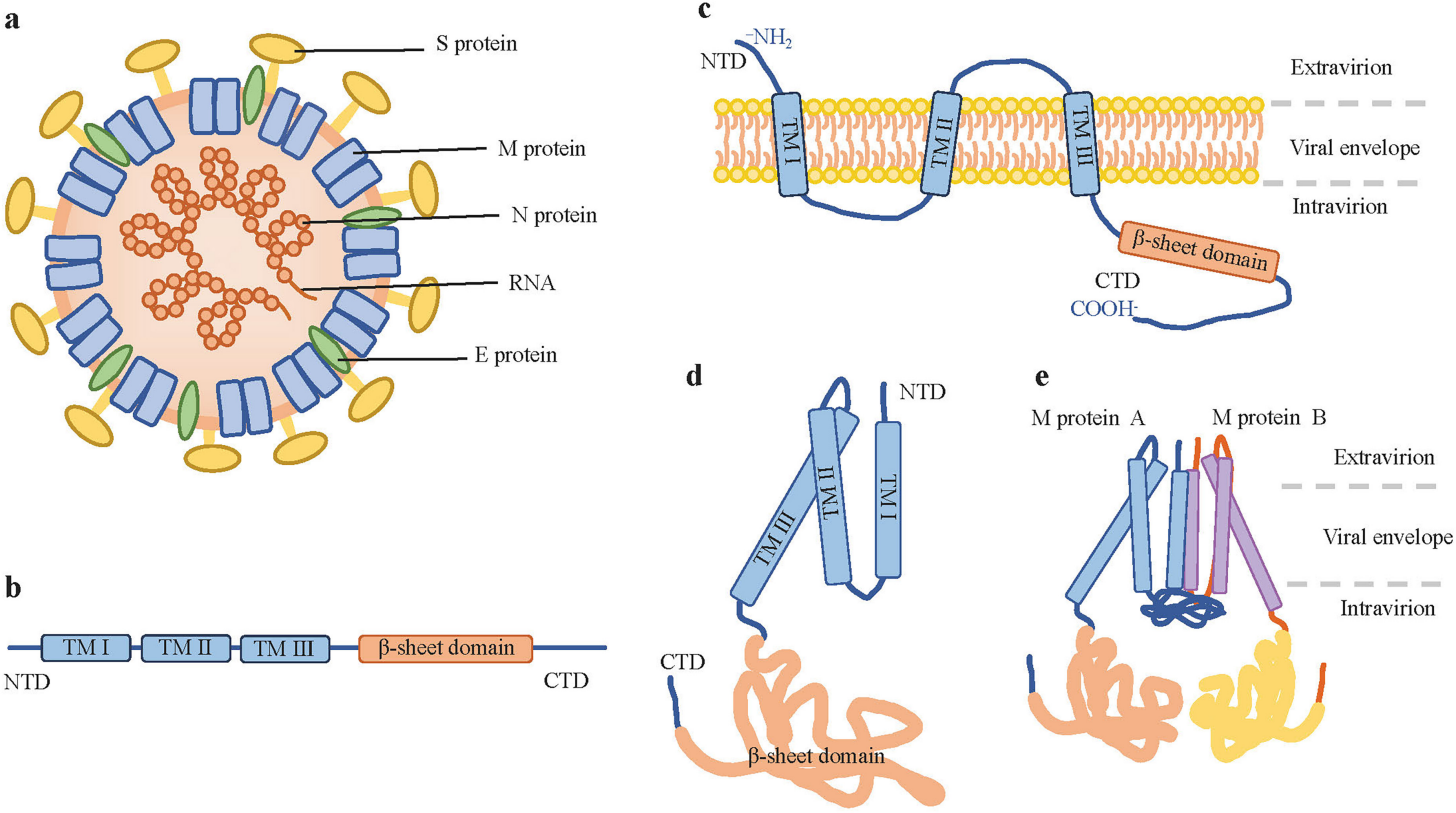
The structure of CoVs and M protein. **(a)** The structure of CoV particle. The M protein is completely embedded in the viral lipid envelope. **(b)** Domain organization of CoV M protein. **(c)** The transmembrane topological structure of the M protein. The CoV M protein spans the viral membrane, with its N-terminal domain located on the exterior of the virion, its C-terminal domain on the interior of the viral membrane, and its transmembrane domain embedded in the lipid envelope. **(d)** M protein monomer. **(e)** The M protein dimers (M–M dimers).

The virus particle of CoV is spherical, with an envelope and spike proteins, and its nucleocapsid exhibits helical symmetry (Fung and Liu, 2019; Lin et al., 2020; Figure 1a). The virus genome is a single-stranded, linear, non-segmented positive-sense RNA, and the genome is formed from 5-capped and 3-polyadenylated, with a size of 25∼30 kb, making it the largest genome among known RNA viruses (Brian and Baric, 2005; Li et al., 2020; Schoeman and Fielding, 2019). The genome contains multiple open reading frames (ORFs) downstream of the 5’ untranslated region (UTR), encoding non-structural precursor polyproteins (Lai et al., 2006; Marra et al., 2003; Park et al., 2012). CoVs have four structural proteins: nucleocapsid protein (N), spike glycoprotein (S), membrane protein (M), and envelope protein (E), which together form the basic structure of the virus (Zandi and Soltani, 2022). Hemagglutinin esterase (HE), a dimer of a class I membrane protein with a molecular mass of 65 kDa, is also present in some closely related β-CoVs, such as Mouse Hepatitis Virus (MHV), Bovine Coronavirus (BCoV) and HCoV-OC43 (Wang Y. et al., 2020), which constitutes short spikes. The ORF3 accessory protein is also found in certain CoVs, including SARS-CoV-2, and has been confirmed to be an important virulence factor (Zhang J. et al., 2022). In all CoVs, the genes encoding the major structural proteins are arranged in the conserved 5’–3’ ordering as S, E, M, and N, within the 3′-proximal one-third of the genome (Brian and Baric, 2005). There are short untranslated regions at both the 5’ and 3’ ends, and the genes encoding putative accessory proteins are located between the M and N proteins (Woo et al., 2012).

The M protein is crucial in the life cycle of CoVs, participating in multiple processes such as viral assembly, envelope formation, and immune modulation. Moreover, it is an important target for therapeutic drug and vaccine development.

## 2 Structural features of M protein

The M protein is the most conserved and abundant structural glycoprotein in the CoV particle and serves as the backbone in viral assembly. Its full length ranges from 217 to 263 amino acids (aa), with a molecular mass of 25–30 kDa (Malik, 2020; Schoeman and Fielding, 2019). In the present study, the M protein of canine coronavirus (CCoV, an α-CoV) is a typical long-sequence representative, comprising 263 aa, whereas the M protein of porcine δ-CoV (PDCoV) is a compact representative, with a length of 217 aa. Although CoVs form their envelope and bud in the endoplasmic reticulum-Golgi intermediate compartment (ERGIC), their viral surface proteins can localize to the compartments downstream in the secretory pathway in viral isolation study (Schoeman and Fielding, 2019). The M protein is mainly located in Golgi apparatus, and it is transported in and out of the organelle via vesicles. It is also located in the endoplasmic reticulum (ER) and the ERGIC (Dong et al., 2016; Nal et al., 2005). This cycling is crucial for its role in recruiting other viral components and facilitating viral assembly.

**FIGURE 2:**
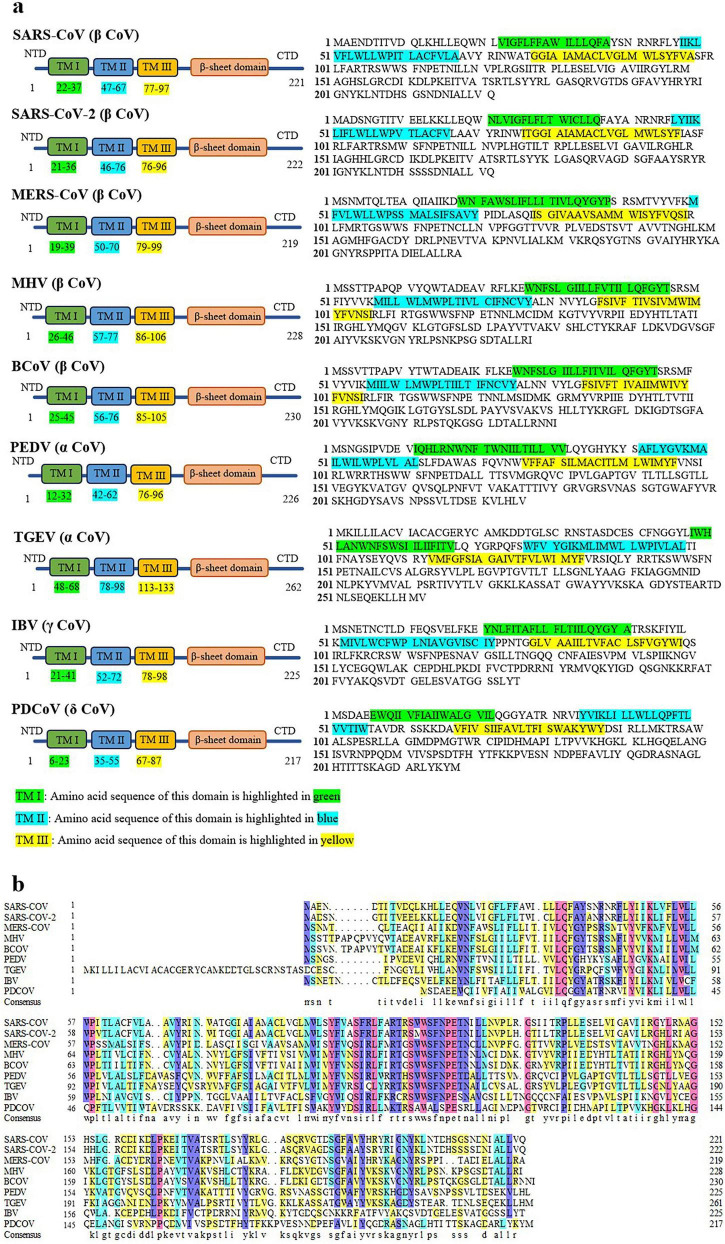
Comparison of the amino acid sequences of the M proteins from several CoVs. **(a)** The structures and sequences of the M protein of SARS-CoV (GenBank: APO40582.1), SARS-CoV-2 (GenBank: QII57163.1), MERS-CoV (GenBank: QGV13486.1), MHV (GenBank: BAJ04700.1), BCoV (GenBank: UZT75403.1), PEDV (GenBank: ANM27773.1), TGEV (GenBank: XEF57925.1), IBV (GenBank: ACS45218.1) and PDCoV (GenBank: QSL97110.1). These sequences were downloaded from the NCBI Protein Database. The analysis data regarding the transmembrane domains are from the UniProt database (https://www.uniprot.org/). **(b)** Multiple sequence alignment of the M proteins was generated using DNAMAN program. Residues with homology levels higher than 33% (yellow), 50% (blue), 75% (violet), and 100% (red) are highlighted.

The M protein is characterized by being embedded in the membrane via three transmembrane domains (Mahtarin et al., 2022; Narayanan et al., 2000; Figures 1b,c). It has been shown that the transmembrane domains of different M proteins contain distinct amino acid sequences (Mahtarin et al., 2022). M proteins from most CoVs exhibit a high degree of structural similarity (Figure 2a). The viruses included in the sequence alignment represent four genera of CoVs (Figure 2b), including the strains that are highly pathogenic to humans and animals. This enables comparative analysis of M protein sequence features across CoV species. Dynamic sequence comparison of the M proteins from nine CoVs reveals that the overall amino acid sequence homology reaches 38.77%. The sequence similarity of the M protein is an important basis for determining the evolutionary relationships of CoVs. The M protein of SARS-CoV-2 shares 90% homology with that of SARS-CoV, and this value is even higher in SARS-CoV strains isolated from animal hosts, such as bats and pangolins (Thomas, 2020). However, it is noteworthy that the similarity of M protein of MERS-CoV is only about 38%.

### 2.1 N-terminal ectodomain

A short N-terminal glycosylated ectodomain—exposed either to the exterior of the virus or to the lumen of intracellular organelles—spans the first 1–19 amino acid residues at the N-terminus. In some α-CoVs, the M protein contains an additional hydrophobic segment that functions as a signal peptide. Research on Transmissible Gastroenteritis Virus (TGEV) has shown that this signal peptide fragment is located within the first 50 amino acids at the N-terminus of the M protein (Escors et al., 2001a). In PDCoV, hydrophobicity analysis reveals that the N-terminal region of the M protein is highly hydrophilic (Wu et al., 2023).

Glycosylation is the process by which sugar chains are covalently attached to specific amino acid residues of proteins via glycosyltransferases, and it is an important post-translational modification. The M protein is always glycosylated at its N-terminal ectodomain (Fung and Liu, 2018). According to the site of modification, it can be classified into N-glycosylation and O-glycosylation. Many experiments have shown that almost all CoV M proteins are exclusively N-linked glycoproteins, especially in α-CoVs and γ-CoVs. After exiting the ER, the M protein reaches the Golgi apparatus, where its N-glycans are further modified (Nal et al., 2005; Perrier et al., 2019). The N-glycosylation of the M protein is not mediated by the virus itself but relies on the host cell’s ER and Golgi apparatus glycosylation modification enzymatic systems operating through the secretory pathway. When M protein mRNA is translated on ribosomes attached to the ER, its N-terminal signal peptide directs the nascent polypeptide into the ER lumen. If the amino acid sequence contains the N-glycosylation consensus sequence (Asn-X-Ser/Thr, where X is any amino acid except proline), the oligosaccharide transferase complex catalyzes the transfer of the core oligosaccharide to the target asparagine residue, initiating glycosylation (Aebi, 2013; Shrimal et al., 2015). Subsequently, glucosidases I and II catalyze the initial processing and folding of the glycoprotein. The glycosylated M protein is then transported to the Golgi apparatus via vesicles, where a series of glycosyltransferases further modify the oligosaccharide chains, ultimately yielding mature, structurally diverse N-linked glycans.

Studies on the N-glycosylation of MERS-CoV and SARS-CoV-2 have revealed that their M proteins are modified with polylactosamine chains, and this modification relies on acidic residues located near the first transmembrane domain (Juckel et al., 2023). During the glycosylation of the M protein, certain acidic residues in its N-terminal region are essential for this modification, in addition to the intrinsic N-glycosylation site. For instance, researchers have confirmed that residues E11, E12, and E18 in the SARS-CoV-2 M protein, a residues E9 and D18 in the MERS-CoV M protein are crucial for the proper glycosylation of these proteins (Juckel et al., 2023). In contrast, the M proteins of certain lineage A β-CoVs such as MHV, BCoV, and HCoV-OC43, are modified by O-linked glycosylation (Liang et al., 2019; Yamada et al., 2000). Studies on MHV have shown that its M protein undergoes O-glycosylation in the Golgi apparatus, with the glycosylation sites located on Ser/Thr residues in the extracellular N-terminal domain (de Haan et al., 2000). Since its identification, O-linked glycosylation has been served as a valuable indicator for investigating the maturation, membrane insertion, and intracellular trafficking of the M protein in these three β-CoVs (Fung and Liu, 2018).

Glycosylation plays a crucial role in maintaining the function of the M protein during viral assembly, release, and interaction membrane interaction. Through deletion mutations of glycosylation sites, it has been found that the N-glycosylation of the SARS-CoV M protein is essential for viral assembly and infectivity. Deletion of this site impairs progeny viral particle assembly (Voss et al., 2006). Deletion mutations of the N3 and N6 glycosylation sites in IBV significantly affect viral virulence (Liang et al., 2019). In addition, the M protein influences viral particle assembly. Substituting O-glycosylation sites in MHV with N-glycosylation sites reduces the efficiency of viral particle recombination (de Haan et al., 2003). Currently, one to four potential N-glycosylation sites have been identified in the M proteins of various CoVs. Generally, most CoV M proteins contain a single N-glycosylation site, and some CoVs have been confirmed to possess multiple glycosylation sites on their M protein (Table 1).

**TABLE 1:** The glycosylation sites of the M proteins of several CoVs.

Virus	Virus species	Number of amino acids	Glycosylation type	Glycosylation site	References
PEDV	Alphacoronavirus porci	226	N-linked	N3, N19 and N189	(Park et al., 2011)
HCoV-NL63	Alphacoronavirus amsterdamense	226	N-linked	N3, N19 and N188	(Dominguez et al., 2012; Müller et al., 2010)
HCoV-229E	Alphacoronavirus chicagoense	225	N-linked	N5, N190 and N207	(Farsani et al., 2012)
HKU2-CoV	Alphacoronavirus rhinolophi	229	N-linked	N6, N22, N192 and N235	(Lau et al., 2007)
HKU4-2-CoV	Betacoronavirus tylonycteridis	219	N-linked	N4 and N20	(Woo et al., 2007)
SARS-CoV	Betacoronavirus pandemicum	221	N-linked	N4	(Voss et al., 2009)
SARS-CoV-2	Betacoronavirus pandemicum	222	N-linked	N5	(Zhang Z. et al., 2022; Schwarze et al., 2024)
MERS-CoV	Betacoronavirus cameli	219	N-linked	N3	(Juckel et al., 2023)
IBV	Gammacoronavirus galli	225	N-linked	N3 and N6	(Zhao et al., 2014)
MHV	Betacoronavirus gravedinis	228	O-linked	T5	(de Haan et al., 2003)
BCoV	Betacoronavirus gravedinis	230	O-linked	S2, S3, T5 and T6	(Ko et al., 2006)
HCoV-OC43	Betacoronavirus gravedinis	230	O-linked	S2, S3, T5, T6, T14 and S28	(Mounir and Talbot, 1992)

### 2.2 Transmembrane domain

The transmembrane domain consists of three transmembrane (TM) helices that anchor the protein in the viral envelope and are labeled TM1, TM2, and TM3 from the N-terminal (Ujike and Taguchi, 2015). The three transmembrane domains of the CoV M proteins are critically important for virus to target the plasma membrane. Moreover, the 50 amino acid residues at the amino terminus containing the TM1 domain, are crucial for achieving effective M–M protein interaction (Tseng et al., 2010). The plasma membrane trafficking signal of the TM3 domain of the SARS-CoV M protein contains the highly conserved residues phenylalanine 95 (F95) and serine 110 (S110), which are essential for viral assembly.

### 2.3 C-terminal intracellular domain

There is a larger C-terminal intracellular structural domain in the M protein, featuring a prominent cytoplasmic tail approximately 6–8 nm in length, which has been identified as the major functional region for interactions between host cell components and other viral proteins (Hsieh et al., 2008). The C-terminal domain, which follows the TM3 region, is organized into a tightly membrane-associated amphipathic structural domain and a distal short hydrophobic structural domain (Ujike and Taguchi, 2015), resulting in a topology comprising both the N-terminal ectodomain and C-terminal cytoplasmic domain (Voss et al., 2009). When investigating the intracellular trafficking of the MERS-CoV M protein, researchers have identified a region in the cytoplasmic tail of its C-terminal domain as the determinant for localization to the trans-Golgi network (TGN). This localization process is mediated by a specific set of four residues (Lys199, Gly201, Tyr203, and Arg204) within the MERS-CoV M protein (Perrier et al., 2019). Furthermore, the presence of the C-terminal domain of MERS-CoV M protein is important for inducing its specific localization.

**FIGURE 3:**
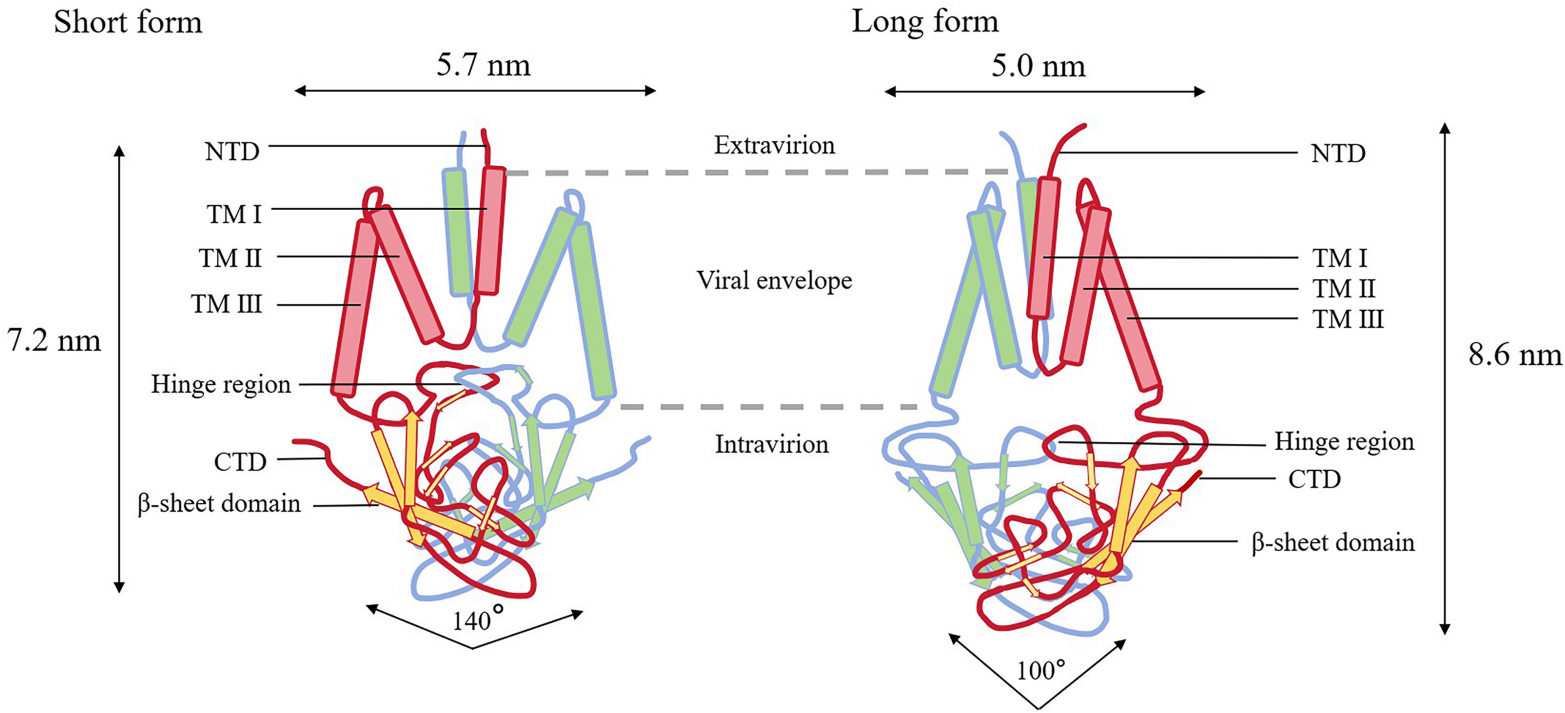
The two conformations of M protein. The M-short dimer is approximately 7.2 nm high and 5.7 nm wide, with a larger upper cavity, a deeper hinge-region insertion, and a BD opening angle of about 140°. The M-long dimer is about 8.6 nm high and 5.0 nm wide, with a smaller upper cavity, a shallower hinge-region insertion, and a narrower BD opening angle of around 100.

The M protein in the virion constitutes the majority of the viral envelope, and the overall scaffold of the envelope is formed by M–M interactions (Arndt et al., 2010; Neuman et al., 2011; Rodríguez-Enríquez et al., 2022; Figures 1d,e). Research indicates that the M protein, which exists as a dimer, adopts two distinct conformations. This structural flexibility enables it to facilitate membrane bending and bind to the nucleocapsid (Rodríguez-Enríquez et al., 2022). The two conformations of the M protein are termed the long and compact (or short) forms (Figure 3), which influence the curvature of the viral envelope. The long form (M-long) confers rigidity, uniformity, and a narrow curvature to the viral envelope, whereas the short form (M-short) plays a contrasting role by providing greater flexibility and reducing spike density (Neuman et al., 2011). Both the long and short forms of the M protein are critical for viral assembly. The M protein forms dimers through interactions with corresponding regions of its structural domains, and in this binding process, specific sites in the three-transmembrane domain play a key role. These dimerizations and subsequent polymerizations are conserved processes. The dimerization of the M protein is a cooperative process involving its transmembrane helices and a C-terminal β-sandwich domain (BD) oriented toward the interior. The dimerization gives rise to a dome-like structure that covers the inner surface of the transmembrane region via the internal sheets of the BD (Zhang Z. et al., 2022). The M protein is structurally similar to the ion channel protein ORF3a, but it does not function as an ion channel by itself. In the two conformations of the M protein dimer (long form and short form), the internal conformation of each monomer remains essentially unchanged, whereas the relative arrangement between the two BDs undergoes significant changes. During the conversion from the long form to the short form, the two monomers approach each other on the extracellular side and separate on the cytoplasmic side. Concurrently, the dimerization interface between the two BDs also undergoes rearrangement, resulting in an increase in the opening angle of the dome-like BD structure from approximately 100–140 (Zhang Z. et al., 2022). The interconversion between the two conformations is regulated by the hinge region, which may be closely linked to modulation of membrane environment, lipid binding, and membrane curvature during viral assembly.

## 3 Biological functions of M protein

### 3.1 The M protein forms the basic structure of the virus

The lattice-like, densely packed matrix structure of the viral envelope results in the lateral interactions of homotypic M–M dimers, which constitutes the fundamental structural components of the viral structure formed by the M protein (de Haan et al., 2000). In SARS-CoV-2, the dimer interface composes 38 residues: 17 are from one M protein molecule (W55, P59, L62, V66, A69, V70, W75, I82, A85, W92, L93, F96, F100, F103, R107, M109, and F112) and 21 are from the other M protein molecule (P59, L62, V66, A69, V70, Y71, I82, A85, W92, L93, F96, I97, F100, F103, A104, R107, S108, M109, S111, and F112) (Marques-Pereira et al., 2022). Experiments on SARS-CoV show that residues W19, W57, P58, W91, Y94, F95, and C158 are essential for M protein homodimer interaction (Tseng et al., 2013). In this context, some host membrane proteins are excluded from the viral envelope through the interaction network formed by M–M dimers. Certain residues of the M protein (K14, Y39, R42, N43, R44, F45, Y71, R72, W75, S94, R101, R107, W110, S173, and R174) participate in the formation of polar, contacting with membrane lipids (Marques-Pereira et al., 2022). SARS-CoV may rely more on cysteine-mediated disulfide bonds to stabilize the dimer, whereas SARS-CoV-2 depends more on electrostatic interactions (Table 2). Hydrophobic interactions underpin M protein membrane intercalation and dimerization across all CoVs. A more stable M protein dimer may enhance the virus’s environment tolerance, improve assembly efficiency, and promote immune escape, thereby increasing transmissibility and pathogenicity. However, the specific mechanism requires further validation through *in vivo* experiments.

**TABLE 2:** The bonding mechanism underlying M protein dimerization in SARS-CoV-2 and SARS-CoV.

Virus	Bonding mechanism	Residues	Characteristic
SARS-CoV-2	Hydrophobic effect	W55, V66, V70, W75, I82, W92, F96, F100, F103, and F112	Primarily mediated by hydrophobic and electrostatic interactions
	Polarity effect	R107	
SARS-CoV	Hydrophobic effect	W19, W57, W91, and F95	Cysteine-mediated covalent bonds are possible, but the hydrophobic effect remains the primary form of dimerization
	Covalent bond (disulfide bond)	C63, C85, C158	

The reticular matrix serves as a scaffold for the assembly of either the E or the N protein. When interacting with the N protein, the M protein packages ribonucleoproteins (RNPs, composed of the viral RNA genome and the N protein) into virions, and this function primarily relies on the intracellular C-terminal domain of the M protein. Moreover, M proteins interact pairwise to form dimers, constituting the viral envelope scaffold; such specific interactions can also incorporate the S or HE protein into lattice vacancies, positioning these proteins regularly within the lattice (Ujike and Taguchi, 2015). Research indicates that it is the collective interactions among M protein dimers that primarily induce membrane curvature, whereas E protein pentamers affect membrane deformation, prevent curvature, and maintain membrane flatness (Collins et al., 2021; Monje-Galvan and Voth, 2021).

### 3.2 The M protein promotes the assembly and release of virus particles

**FIGURE 4:**
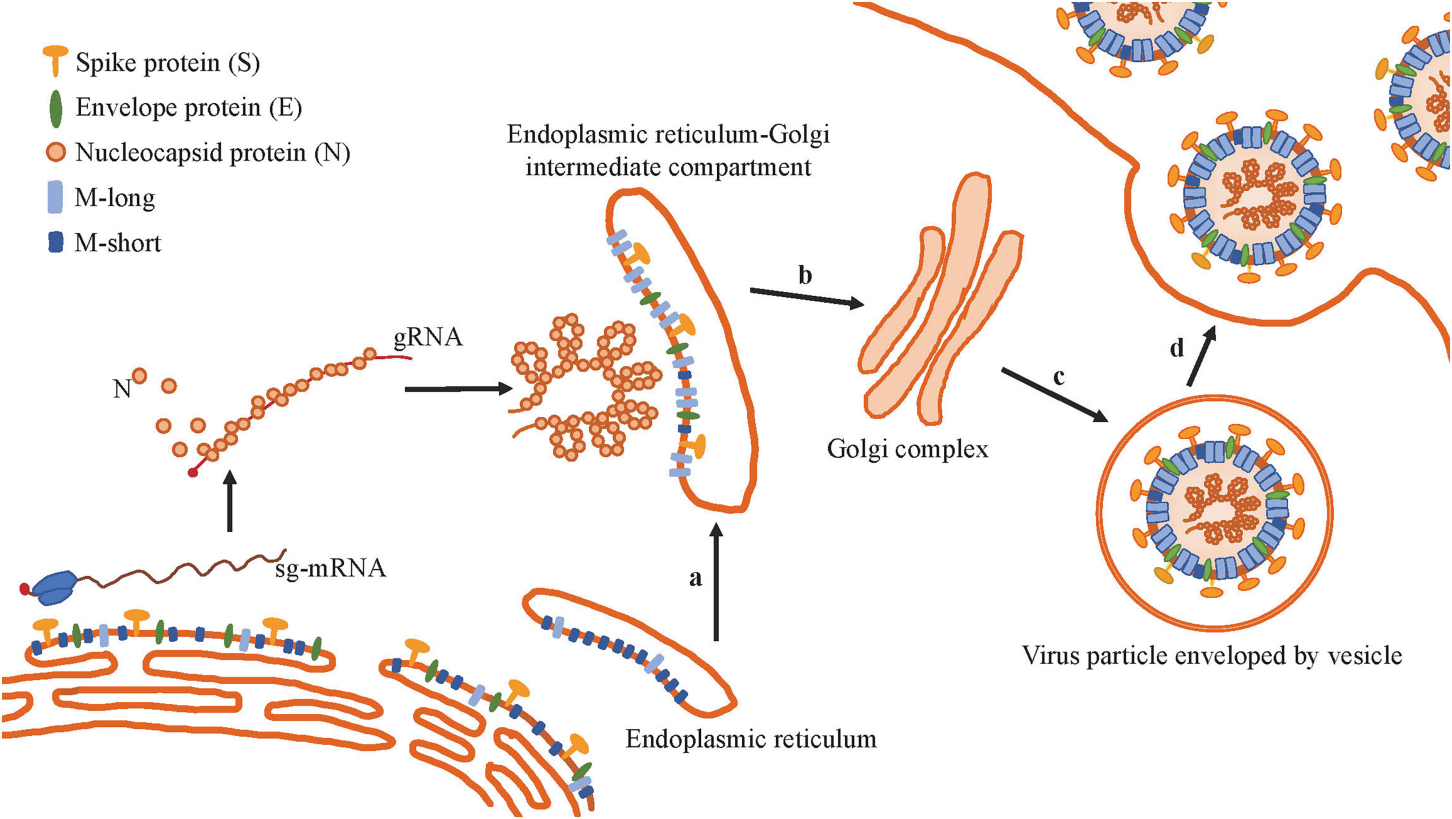
M protein promotes the assembly and release of virus particles. **(a)** On the ERGIC membrane, the number of M-short proteins exceeds that of M-long proteins. Recruitment of the S, E proteins, the RNP, as well as conversion of M-short to M-long. **(b)** The ERGIC associates with the RNP and then traffics to the Golgi complex. **(c)** Viral maturation, budding, and assembly. **(d)** Release. The virus is released outside the cell via exocytosis.

The CoV genomes replicate in the cytoplasm and assemble into viral particles by budding into the smooth membrane of the ERGIC, then traffic to the ER, and are released via exocytosis to infect surrounding cells (Liang et al., 2019; Steiner et al., 2024; Figure 4). The M protein of CoVs is a central player in virion assembly, particularly during replication and transcription, serving as the key organizer that transforms host cell membranes into sites for viral particle production (Bracquemond and Muriaux, 2021; Kuo et al., 2016a; Steiner et al., 2024). The M protein dynamically cycles between the Golgi complex and the ER, and returns to the Golgi from the plasma membrane via the endocytic recycling pathway. The synthesis of the M protein increases during CoV infection (Wang R. et al., 2020). Immunofluorescence and Western blot analyses reveal that M protein expression can be detected as early as post-infection and persists throughout the course of infection until cell death (Wu et al., 2023). CoV assembly is critically dependent on the proper and accurate intracellular transport of the M protein (Desmarets et al., 2023). Through M–M interactions, a scaffold for viral assembly is formed, and host cell membrane proteins are excluded, thereby conferring specificity to the viral envelope (Liang et al., 2019). Heterotypic interactions facilitate the recruitment of other structural proteins and the viral genomic RNA to the assembly site. The M protein not only self-associates but also assists in virion assembly and interacts with other structural proteins (N, E, and S), as well as with accessory proteins (including ORF3), host proteins, and non-structural proteins (Mahtarin et al., 2022; Rodríguez-Enríquez et al., 2022). By recruiting other structural proteins to the budding site, the M protein organizes a specific membrane structural domain for viral assembly (Neuman et al., 2011). Through these interactions, the M protein ensures the efficient assembly and release of new viral particles (Castaño et al., 2021).

#### 3.2.1 M protein interacts with E protein to form virus-like particles and promote virion release

The co-localization and interaction of the M and E proteins represent one of the most mature and well-defined protein-protein interactions among the structural proteins of CoVs, which are crucial for the formation of CoV protein-containing vesicles (Lopez et al., 2008; Swann et al., 2020; Xu et al., 2020). The coexpression of M and E proteins can induce the formation of virus-like particles (VLPs) and their release. Mediated by the C-terminal domains of the two partner proteins, this interaction occurs at the cytoplasmic side of the ERGIC. Studies have shown that deletions of C-terminal in both the M and E proteins of SARS-CoV-2 impair their ability to interact (Mukherjee et al., 2020), whereas ubiquitination of the N-terminal domain of M protein stabilizes the M-E interaction, indicating a functional connection between the C-terminal and N-terminal domains of the M protein. Recently, the interaction between the M and E proteins of SARS-CoV-2 has been clarified: it reveals that the M-E protein interaction mediates vesicle release by enhancing M protein self-interaction (Satarker and Nampoothiri, 2020; Zhou et al., 2023). This observation may relate to the role of the M protein in viral assembly and/or the role of the E protein in membrane scission (Bracquemond and Muriaux, 2021). Current findings propose that the E protein does not directly induce membrane curvature but rather enhances M protein self-interaction (Yuan et al., 2021). This enhanced interaction indirectly facilitates virion budding by increasing membrane fluidity. Due to the presence of M protein, viruses and VLPs have a thicker envelope region than vesicles or exosomes. However, the formation of VLPs strictly requires the coexpression of the M and E proteins, as the expression of M protein alone is insufficient. Functional cooperation between the M and E proteins leads to the formation of pseudo particles, which exhibits interfering activity similar to that of intact virions (Song and Park, 2012). For ensuring formation of uniformed size of viral particles, the M-E complex is essential for viral maturation and ultimately enables viral release (Yuan et al., 2021).

#### 3.2.2 The M protein interacts with S protein and stabilizes the S protein in assembling virus particle

The M protein dimer is responsible, in part, for recruitment of the S protein. Functional experiments in MHV and BCoV have demonstrated that, in the VLP assembly system, coexpression of the M and S proteins significantly enhances S protein incorporation efficiency and improves its structural orderliness (Ujike and Taguchi, 2015); whereas disruption of M protein dimerization leads to defective S protein recruitment. When coexpressed with the E protein, the M protein participates in regulating S protein localization, likely at the ERGIC or cis-Golgi assembly sites. This intracellular retention of S protein inhibits cell–cell fusion, thereby preventing the formation of syncytia (Boson et al., 2021). In fact, the M protein preferentially captures the S protein and incorporates it into virions or VLPs. The M protein mediates intracellular retention of the S protein by directly binding to its cytoplasmic tail. Moreover, the M protein interacts with the S protein and retains it in the Golgi/ERGIC compartment, where the majority of viral assembly takes place (Arndt et al., 2010; Fung and Liu, 2018). Heparan sulfate proteoglycans (HSPGs) are glycoproteins that constitute ubiquitous components of the mammalian cell surface. The M protein of HCoV-NL63, in synergy with the S protein, facilitates host cell invasion by promoting the binding to HSPGs containing heparan sulfate (Naskalska et al., 2019).

#### 3.2.3 The M protein interacts with the N protein and facilitates viral infection

During virus assembly, the interaction between the M and N proteins plays an irreplaceable role. Various studies have shown that this interaction recruits the RNP complex synthesized in the cytoplasm to the viral assembly site (primarily the ERGIC membrane). In most CoVs, the primary region mediating the M–N protein interaction is localized in the cytoplasmic domain of the C-terminal region of both the M and N proteins. A study of SARS-CoV has shown that the leucine motif (218LL219) at the C-terminus of the internal domain of M protein is essential for efficient packaging of the N protein into VLPs (Tseng et al., 2013). In β-CoVs, research provides direct evidence that the M and N proteins interact in MHV-infected cells, as demonstrated by reciprocal co-immunoprecipitation using an anti-M monoclonal antibody. The main determinant of the MHV M–N protein interaction is located in domain 3 of C-terminus of the N protein (Hurst et al., 2009). In SARS-CoV-2, the mature nucleocapsid (a single-genome condensate) directly interacts with the M protein (Cubuk et al., 2021). Research on SARS-CoV-2 indicates that the interaction between the N and M proteins during the budding process is critically dependent on the C-terminal domain of the N protein (Han et al., 2024). This interaction represents a critical step in virion assembly, bridging the packaged genome to the viral envelope (Bai et al., 2021). In α-CoVs, research on TGEV shows that the C-terminal region of the M protein interacts with the N protein (Escors et al., 2001b). The TGEV M–N protein interaction is temperature-sensitive, and the binding is more stable at lower temperatures. Moreover, assembly of the M and N proteins also requires the assistance of the E protein to enhance interaction stability. Disruption of the M–N interaction severely impairs or completely blocks VLP formation and infectious virion production, findings consistently confirmed across all CoV studies.

The above facts demonstrate that coexpression of the M protein with other structural proteins leads to robust VLP production. Further evidence shows that the SARS-CoV-2 M protein promotes viral replication by disrupting STX18 to induce ATG14-mediated lipid phagocytosis and by combining with its ability to degrade RSAD2 (Yuan and Ding, 2024; Yuan et al., 2024).

### 3.3 The M protein participates in the immune response

#### 3.3.1 Altering the expression of IFN and regulate the innate immune response

The M protein can alter IFN expression and regulate the innate immune response (Table 3). Glycosylation of N-terminal extracellular domain of the M protein is not indispensable for CoV replication, but it does play a role in virus–host interaction as well as in viral pathogenesis (Liang et al., 2019). Studies have found that, in infectious bronchitis virus (IBV), N-linked glycosylation of the M protein may promote apoptosis and proinflammatory responses during CoV infection, potentially by modulating ER stress and subsequently triggering proinflammatory cytokines production. A study using recombinant MHV to change the glycosylation of its M protein from O-linked to N-linked found that the type I interferon levels were higher than those of wild-type MHV after the modification (Fung and Liu, 2018). This result demonstrates that the glycosylation status of the M protein can modulate the host innate immune response. Additionally, in MHV, researchers have demonstrated that the glycosylation state of the M protein critically influences the type I interferon (IFN) induction *in vitro* (de Haan et al., 2003), whereas N-linked glycosylation of the M protein’s oligosaccharide side chains has a stronger interferon-inducing capacity than O-linked glycosylation.

**TABLE 3:** Key Structure and mechanism of pathogenic HCoV M protein in regulating IFN.

Virus	Regulation of IFN	Key structure	Mechanism	References
SARS-CoV	Upregulate	Val68	Acts as an intracellular PAMP to activate IFN-β signaling pathway	(Wang and Liu, 2016)
	Inhibit	TM domains	Inhibits the formation of the TRAF3-TBK1 complex to prevent the activation of IRF3	(Kindler et al., 2016)
		The entire M protein	Impedes the formation of the TRAF3-TANK-TBK1/IKKε complex	(Siu et al., 2009)
SARS-CoV-2	Inhibit	TM domains	Inhibits the aggregation formation of MAVS, thereby preventing the recruitment of downstream signaling molecules such as TRAF3, TBK1 and RIG-I, and further inhibiting the phosphorylation and nuclear translocation of IRF3	(Fu et al., 2021; Zheng et al., 2020)
		Trp31 and Leu34	Induces mitochondrial autophagy to disrupt the MAVS-mediated type I IFN signaling pathway	(Hui et al., 2021)
MERS-CoV	Inhibit	TM1 domain	Binds to TRAF3 through its N-terminal transmembrane domain, thereby disrupting the formation of the TRAF3-TBK1 complex, suppresses the phosphorylation and activation of IRF3	(Lui et al., 2016)
		The entire M protein	Cooperates with ORF4a and ORF4b to inhibit the activation of interferon-stimulated response element (ISRE)	(Yang et al., 2013)

Certain CoVs can upregulate IFN expression during host infection. Studies on SARS-CoV have shown that the SARS-CoV M protein can upregulate the transcription of IFN-β (Wang and Liu, 2016). The M protein directly acts as a pathogen-associated molecular pattern (PAMP) in the cytoplasm to activate the Toll-like receptor signaling pathway and the TBK1–IRF3 signaling cascade, thereby stimulating IFN-β expression. In addition, high levels of IFN-α can be detected in the host during early TGEV infection, and the M protein has been confirmed as a potential IFN inducer that primarily promotes the activation of IRF3 (Zhou et al., 2017).

In some CoV infections, the M protein has been shown to play a role in inhibiting IFN expression. The SARS-CoV-2 M protein is a negative regulator of the innate immune response (Fu et al., 2021). SARS-CoV-2 suppresses the type I IFN response by promoting autophagy. This is achieved by inducing mitophagy through the M protein, which specifically inhibits RIG-I-MAVS-triggered IFN-β signaling (Hui et al., 2021). Additionally, the SARS-CoV-2 M protein inhibits the innate antiviral immune response triggered by RNA viruses by impairing recruitment of the mitochondrial antiviral signaling (MAVS) complex by TANK-binding kinase 1 (TBK1), TNF receptor-associated factor 3 (TRAF3), and interferon regulatory factor 3 (IRF3). The M protein targets MAVS to inhibit the innate antiviral response, and its TM1 and TM2 domains are critical for this inhibitory function. Moreover, the SARS-CoV-2 M protein can antagonize both type I and III IFNs, as it can affect the formation and assembly of a multiprotein complex (the RIG-I/MDA-5-MAVS-TRAF3-TBK1 signalosome) (Zhang et al., 2021; Zheng et al., 2020). MERS-CoV utilizes its M protein to suppress the expression of type I IFN by inhibiting TBK1-dependent phosphorylation and activation of IRF3, thus evading the host innate antiviral response (Kindler et al., 2016; Lui et al., 2016). Additionally, SARS-CoV also suppresses type I IFN production by disrupting the TRAF3-TBK1 association via interaction with TRAF3 (Siu et al., 2009). A study has found that the PEDV M protein inhibits TBK1/IKKε-induced phosphorylation and dimerization of IRF7, thereby suppressing IFN-I production and enhancing viral replication (Li et al., 2021).

Different human coronaviruses (HCoVs) cause infections in hosts with markedly varying severity. Pathogenic HCoVs. (SARS-CoV, SARS-CoV-2, and MERS-CoV) can evade innate immune responses by modulating the type I IFN pathway, frequently resulting in severe lower respiratory tract diseases such as acute respiratory distress syndrome (ARDS) (Table 2). During these highly pathogenic HCoV infections, upregulation of inflammatory cytokine genes often leads to dysregulation of the inflammatory response. In contrast, non-pathogenic HCoVs (HCoV-229E, HCoV-OC43) exhibit a comparatively weaker IFN-inhibitory capacity and typically cause only mild upper respiratory tract infections, commonly known as the common cold. Highly pathogenic HCoVs can upregulate IFN-stimulated genes, but in these viruses, the M protein has IFN-antagonistic activity and inhibits IFN expression. A reasonable explanation for this apparent contradiction is that the M protein itself can stimulate the production of IFN. However, when the intracellular IFN I pathway is activated by other pathways, the M protein exerts a negative regulatory effect on activated IFN I pathway. Furthermore, the differences in interferon regulation may also be related to other viral proteins, such as accessory ORFs, NSP1, and NSP2 (Liu et al., 2023b). Interferon regulation by CoV M proteins varies across strains, indicating that IFN regulation by M proteins is not evolutionarily conserved among HCoVs (Zheng et al., 2020).

#### 3.3.2 M protein interacts with heat shock protein and enhances viral infection

Studies on porcine epidemic diarrhea virus (PEDV) have found that the M protein plays a key role in the collaborative replication of PEDV (Park et al., 2021). The infection is accompanied by strong overexpression of heat shock protein 70 (HSP70), which increases the replication speed of PEDV and leads to production of more virions. Through its direct interaction with HSP70, the M protein enhances the expression of this host factor, thereby facilitating PEDV replication. Another study also indicates that the PEDV M protein, which is distributed throughout the cell, affects the growth of intestinal epithelial cells (IECs) (Xu et al., 2015). It is also worth mentioning that previous studies have confirmed that the PEDV M protein, in addition to playing an important role in the viral assembly process, can induce complement-dependent antibodies that neutralize the virus (Song and Park, 2012). Studies on transmissible gastroenteritis virus (TGEV) have elucidated a previously unrecognized function of the M protein, demonstrating its participation in the initial stages of viral replication (Ji et al., 2023). The interaction between heat shock cognate protein 70 (HSC70) and the M protein intracellular domain, mediated by the substrate-binding domain (SBD) of HSC70, plays a role in viral entry. The M protein orchestrates internalization of TGEV by interacting with the host factor HSC70, thereby directing the virion into the cells via the clathrin-mediated endocytosis (CME) pathway. In human and mouse CoV-induced fulminant viral hepatitis, the expression of HSP70 is distinctly upregulated. These findings suggest that HSP70 enhances the replication of CoV in hepatocytes and further facilitates the release of pro-inflammatory cytokines such as IL-1β, TNF-α, and IL-6 during infection (Wang Q. et al., 2024). Differential gene expression analysis has revealed that the SARS-CoV-2 M protein significantly upregulates HSPs and co-chaperone proteins, and this regulatory effect is stronger than that of the N protein (Albalawi et al., 2025). Notably, HSPA6, HSPA1B, HSPBP1, and HSPH1 were identified as HSPs with increased expression (Albalawi et al., 2025; Sun et al., 2021). Severe SARS-CoV-2 infection can trigger an excessive immune response, resulting in a “cytokine storm,” in which macrophages play a central role, and HSP70 further promotes the infiltration of mononuclear phagocytes and neutrophils into the liver after CoV infection (Wang Q. et al., 2024).

#### 3.3.3 M protein mediates the humoral and cellular immunity

It has been proven that the SARS-CoV M protein can induce strong neutralizing antibodies (Pang et al., 2004). Two immunodominant epitopes of the SARS-CoV M protein have been identified in the extreme N-terminal and C-terminal regions (He et al., 2005). The T cell epitope cluster, which is contained in the transmembrane domain of the M protein, plays a major role in driving M protein specific cellular immunity (Liu et al., 2010), inducing CD8 + T-cell responses and eliciting dominant cellular immunity during SARS-CoV infection (Ng et al., 2016; Tang et al., 2011). An immunodominant B cell epitope is located at the S4 position of the active center of the SARS-CoV-2 M protein, which potentially triggers neutralizing antibodies that inhibit the protease function of the M protein (Lu et al., 2021). In a study of PEDV, seven linear B-cell epitopes (designated M1 through M7) have been identified on the M protein, with lengths ranging from 12 to 22 aa (Polyiam et al., 2022). All seven peptide segments react positively with PEDV-seropositive porcine serum. Notably, epitopes M1, M2, and M6 are able to inhibit the neutralizing activity of all tested sera and are regarded as the most promising neutralizing targets in the M protein.

### 3.4 M protein affects the function of mitochondria and induces apoptosis

The M protein triggers caspase-dependent apoptosis via the mitochondrion-mediated pathway. Specifically, in SARS-CoV, the M protein promotes apoptosis by modulating the release of mitochondrial cytochrome c and affecting the cellular Akt pro-survival signaling pathway (Chan et al., 2007). Akt, also known as protein kinase B (PKB), is a central player in the cellular kinase cascade, regulating diverse cellular functions and influencing the viral life cycle. The PKB/Akt pathway is involved in multiple viral infections, including but not limited to SARS-CoV (Mizutani et al., 2006a; Mizutani et al., 2006b). Caspase-9 is a target of PKB/Akt-mediated phosphorylation, and the activity of caspase-8 is also indirectly regulated by PKB/Akt. Overexpression of the M protein suppresses PKB/Akt phosphorylation, leading to downregulation of pro-survival signaling and ultimately triggering apoptotic cell death. The PKB/Akt signaling pathway plays a pivotal role, as it is critical for maximal viral production and apoptosis regulation. Through the PKB/Akt signaling cascade, the expression of M protein activates caspase-8 and caspase-9 (Tsoi et al., 2014).

Depending on caspase-9, transient overexpression of the SARS-CoV-2 M protein initiates mitochondrial apoptosis by engaging B-cell lymphoma 2 (BCL-2) ovarian killer (BOK). According to the studies on SARS-CoV-2, the M protein has been identified as an inducer of mitochondrial apoptosis in pulmonary epithelial cells (Yang et al., 2022). The M protein triggers caspase-associated cell death, leading to the increased pulmonary barrier permeability (Wang F. et al., 2024; Yang et al., 2022). Studies investigating the interaction between SARS-CoV-2 M protein and human proliferating cell nuclear antigen (PCNA) have shown that the M protein induces cytoplasmic translocation of PCNA from the nucleus. This finding suggests that the M protein may promote DNA damage by involving PCNA (Zambalde et al., 2022).

A study has demonstrated that the M protein of SARS-CoV-2 induces neurodegeneration by disrupting the interaction between the Golgi apparatus and mitochondria (Wang F. et al., 2024). This study also confirms that the M protein interacts with two regulatory factors of the Arf signaling pathway, ArfGEF1 and ArfGAP1, in the Golgi apparatus. Since the PI4KIIIβ/Arf1 pathway is involved in the interaction between the Golgi complex and mitochondria, expression of the M protein may disrupt Golgi structure, thereby leading to structural abnormalities and functional impairment of mitochondria. The M protein promotes retrograde transport from the Golgi apparatus to the ER, inducing mitochondrial fragmentation and functional impairment, which results in decreased ATP production, excess reactive oxygen species (ROS), and ultimately cell death. Additionally, the autophagy cargo receptor (ACR) SQSTM1 plays a dual role in host-pathogen interactions and is often context-dependent. By phosphorylating SQSTM1 at a special site, the M protein promotes antiviral activity (Li et al., 2024). Another study shows that the M protein can also mediate and promote mitophagy. M protein contains a β_3–5_ domain, which plays a significant role in promoting mitochondrial autophagy through PDPK1-mediated phosphorylation of SQSTM1 site. The β_3–5_ domain can counteract the mitophagy promoted by the M protein. The M interferes with the function and distribution of the Golgi apparatus, leading to mitochondrial abnormalities and dysfunction, thereby contributing to neurodegeneration.

### 3.5 M protein interacts with viral RNA and packages the RNA into the viral particle

Genome packaging is a critical process arising from the parasitic status of CoV (Li et al., 2024). During the replication process of the CoV, large amounts of RNA are produced, including positive-strand genomic RNA (gRNA), negative-strand genomic RNA and positive-strand subgenomic RNA (Narayanan et al., 2000). Both the M and N proteins are known viral molecular partners that mediate packaging signal (PS) recognition. To ensure selective packaging of gRNA into viral particles, the CoV distinguishes its full-length gRNA from other cellular RNAs through genome-specific PS and specifically recruits M and N proteins (Bai et al., 2021). There are three types of models describing how the PS, N, and M proteins interact with each other to help CoVs achieve packaging selectivity. The N protein of the CoV contains two highly conserved, independently folded domains (the N-terminal domain and the C-terminal domain), and a third, predominantly acidic C-terminal region termed the N3 domain (Zhao et al., 2024). The genetic information model integrates the known functions of the N protein domain. In this model, the CTD serves as an RNA-binding module, whereas the N3 domain is hypothesized to be the exclusive site for interaction with the M protein (Chang et al., 2014; Kuo and Masters, 2013; Kuo et al., 2014; Kuo et al., 2016b). The second model explains how the M protein primarily recognizes PS. Evidence indicates that the M protein is the primary and perhaps the exclusive PS recognition element (Masters, 2019). The internal domain of the M protein is oligomerized at the assembly site of the cell membrane, enabling it to mediate specific PS recognition. This implies that each assembled virion contains an individual and specific contact site between the M protein and the gRNA PS (Neuman et al., 2011). Therefore, the M protein drives their interaction and condensation by initiating the nucleation of M-N and N-N protein complexes. The presence of C-tail is vital for the effective encapsidation of gRNA by the N protein, and this process is regulated by M protein (Han et al., 2024).

Studies on MHV have demonstrated that the M protein modulates PS recognition. Evidence indicates that the specific interaction between the M protein and the intracellular defective interfering ribonucleoprotein (DI RNP) complex involves the PS (Narayanan and Makino, 2001). The M protein mediates efficient packaging of DI RNP complexes into MHV particles by specifically recognizing and binding to those complexes that carry packaging signals. Researchers have concluded that the PS is responsible for the specific interaction between the M protein and particular intracellular RNP complexes, thereby drives the efficient and selective packaging of MHV RNAs containing these signals into viral particles.

The M protein plays a significant role in the viral life cycle and also modulates host cells activity. Its functions are closely related to its structural features. The major functions of M protein are elaborated in detail in Table 4.

**TABLE 4:** The major functions of M protein.

Function	Functional particulars		Typical type of virus	References
Constructing the basic structure of the virus	Determining the morphology of the viral envelope		All types of CoVs	(de Haan et al., 2000; Neuman et al., 2011)
	Interacting with the proteins of M, E, N and S			(Collins et al., 2021; Ujike and Taguchi, 2015; Monje-Galvan and Voth, 2021)
Guiding the assembly and release of virus particles	Co-expressing with other structural proteins to forms VLPs		All types of CoVs	(Castaño et al., 2021)
	Interacting with E protein			(Lopez et al., 2008; Satarker and Nampoothiri, 2020; Song and Park, 2012; Swann et al., 2020; Xu et al., 2020; Yuan et al., 2021; Zhou et al., 2023)
	Interacting with S protein			(Boson et al., 2021; Naskalska et al., 2019)
	Interacting with N protein			(Han et al., 2024; Yuan et al., 2024; Yuan and Ding, 2024)
Participating in the immune response	Altering the expression of IFN	Upregulate	SARS-CoV, MHV, IBV, TGEV	(de Haan et al., 2003; Fu et al., 2021; Hui et al., 2021; Kindler et al., 2016; Lui et al., 2016; Wang and Liu, 2016; Zhang et al., 2021; Zheng et al., 2020; Li et al., 2021)
		Inhibit	SARS-CoV, SARS-CoV-2, MERS-CoV, PEDV	
	Interacting with HSP70, enhancing viral replication		PEDV, TGEV, MHV, SARS-CoV-2	(Albalawi et al., 2025; Ji et al., 2023; Park et al., 2021; Sun et al., 2021; Wang Q. et al., 2024; Xu et al., 2015)
	Possessing B cell and T cell antigenic epitopes		SARS-CoV, SARS-CoV-2	(He et al., 2005; Liu et al., 2010; Lu et al., 2021; Ng et al., 2016; Tang et al., 2011)
Inducing apoptosis	Triggering mitochondrial apoptosis		SARS-CoV, SARS-CoV-2	(Hui et al., 2021; Wang F. et al., 2024; Li et al., 2024)
	Interfering with the connection between PDK1 and PKB/Akt, stimulating Caspases-8 and Caspases-9			(Chan et al., 2007; Mizutani et al., 2006a; Mizutani et al., 2006b; Tsoi et al., 2014)
	Leading to neurodegeneration and DNA damage			(Zambalde et al., 2022)
Interacting with viral RNA	Interacting with viral RNA, packaging RNA into virus particles		All types of CoVs	(Bai et al., 2021; Kuo and Masters, 2013; Kuo et al., 2014; Kuo et al., 2016b; Masters, 2019; Narayanan and Makino, 2001; Narayanan et al., 2003; Tsoi et al., 2014; Zhao et al., 2024)

## 4 Application

In terms of the application of M protein, although it is not yet widely used in clinical practice as a therapeutic agent, findings from various studies suggest that the M protein holds promising potential for broad clinical applications.

### 4.1 The important application value in drug research of M protein of CoVs

Due to its crucial role in the viral life cycle, the M protein of CoVs has significant potential for drug development. The M protein plays a key role in the assembly and budding of the virus, and its structure and function are essential for viral maturation and release. Therefore, the M protein can serve as a potential target for the research and development of antiviral drugs, facilitating the creation of specific therapeutic drugs against CoVs and providing robust strategies for epidemic prevention and control. For example, as mentioned above, the PDPK1-targeted peptide effectively eliminates CoV infection by redirecting SQSTM1 from the viral M protein to mitochondria, thereby restoring viral phagocytosis and simultaneously inhibiting mitochondrial autophagy (Li et al., 2024). HSC70 has been identified as a host factor required for the internalization of TGEV, dependent on its interaction with the viral M protein. Consequently, disrupting the M-HSC70 interaction presents a viable strategy for designing antiviral therapies against TGEV (Ji et al., 2023). Through the study of the M protein, researchers have identified a novel drug target within the CoVs replication cycle, along with a potent small-molecule inhibitor. It is essential to consider the membrane environment in drug design when targeting the M protein as a viral drug target. Research has shown that the _199_KxGxYR_204_ motif in the C-terminal tail of the MERS-CoV M protein may represent such a CoV-specific target (Desmarets et al., 2023). Recently, a study has found that a structural expansion below the transmembrane domain and above the β-sheet intercalation domain within the M protein dimer is observed in all M proteins. This site is located in the β-sheet sandwich domain near the C-terminus and may serve as a potential drug-binding site (Yegnaswamy et al., 2025). Apart from the M protein, certain host cell components can also serve as targets for antiviral drug development. Research has found that PEDV, SARS-CoV-2, IBV, and PDCoV can all interact with Aurora A (AurA) and histone deacetylase 6 (HDAC6) in host cells via their M proteins, thereby inducing ciliary disassembly during the early infection (Zhuang et al., 2025). This finding suggests that AurA and HDAC6 may serve as broad-spectrum therapeutic targets for multiple CoV infections.

At present, numerous studies have identified a variety of compounds with potential activity against HCoVs (Table 5). CIM-834, an M-targeting molecule, exerts its late-stage mechanism of action by impeding the conformations interconversion from the M-short form to the M-long form, thereby inhibiting viral particle assembly (Laporte et al., 2025). Meanwhile, JNJ-9676, a small-molecule inhibitor targeting the M protein, exerts its antiviral effect by inducing the formation of a binding pocket and stabilizing a novel conformational state of the M protein (Van Damme et al., 2025). It exhibits potent antiviral activity *in vitro* against zoonotic strains of SARS-CoV and SARS-CoV-2 originating from bats and pangolins. Caffeic acid and ferulic acid are considered to have the potential to inhibit the SARS-CoV-2 M protein, which provides new insights for the development of anti-COVID-19 drugs (Bhowmik et al., 2020). Both compounds exert their effects by binding to Lys50 of the SARS-CoV-2 M protein. Similarly, colchicine, remdesivir, bafilomycin A1, and temozolomide can also bind to the M protein, thereby exerting an inhibitory effect on it (Peele et al., 2021). Additionally, ARF1 small-molecule inhibitors brefeldin A (BFA) and golgicide A (GCA), as well as the synthesized mimic peptide PEP17, can disrupt the localization of ARF1 and M protein on the ERGIC, thereby weakening virion assembly and inhibiting SARS-CoV-2 replication (Zhang et al., 2025). However, the effectiveness of these drugs still needs to be further verified through research.

**TABLE 5:** Current research findings on therapeutic drugs targeting the M protein.

Potential therapeutic drug	Molecular target	Mechanism of action	Possible effects	Virus	References
CIM-834	Pro123 of M protein	Binds to and stabilizes the M protein in its short form, thereby preventing its conformational switch to the long form	Inhibit the viral assembly and reproduction. Exhibits antiviral activity and blocks viral spread	SARS-CoV, SARS-CoV-2	(Laporte et al., 2025)
JNJ-9676	Ser99 and Asn117 of M protein	Stabilizes the M protein dimer in the conformational state intermediate between the long and short forms		SARS-CoV-2 MERS-CoV, HCoV-OC43, MHV	(Van Damme et al., 2025)
Caffeic acid	Lys50 of M protein	Form hydrogen bond with the active site residue of the M protein		SARS-CoV-2	(Bhowmik et al., 2020)
Ferulic acid	Lys50 of M protein				
Colchicine	Met109 of M protein				(Peele et al., 2021)
Remdesivir	Ala40 and Arg131 of M protein				
Bafilomycin A1	Ala40, Asn41 and Asn43 of M protein				
Temozolomide	Asn41, Asn113 and Glu115 of M protein				
Brefeldin A (BFA)	ARF1	Disrupt ARF1 and M localization at the ERGIC and abate virion assembly		SARS-CoV-2	(Laporte et al., 2025)
Golgicide A (GCA)	ARF1				
PEP17	Conformational epitopes of M protein	Blocks the binding of M protein to ARF1 through competitive binding			

### 4.2 M protein can serve as an immunological target for developing assays to detect the CoVs

As one of the viral structural proteins, the biochemical and immunological properties of the M protein indicate that it is highly suitable as a specific antigen for detecting CoV infection (Wu et al., 2023). The M protein can be used to develop immunoassay methods, such as enzyme-linked immunosorbent assay (ELISA) and immunochromatographic assays. In CCoV detection, ELISA based on recombinant M protein is regarded as an effective method for detecting CCoV-specific antibodies in canine serum (Elia et al., 2003). Similarly, an ELISA based on the M protein is highly significant for detecting PEDV and distinguishing its infection from other related porcine viral infections (Ren et al., 2011). In the research of TGEV, the M protein has been expressed in *Escherichia coli* as a GST fusion protein. Using the phage clone phTGEV-M7 as the antigen, a phage-based immunosorbent assay (PHAGE-ELISA) was established, which not only distinguishes TGEV from other CoVs., but also exhibits higher sensitivity than the conventional antibody-based ELISA (Zou et al., 2013). The M gene sequences of different PDCoV strains share 99% nucleotide identity, and are therefore commonly used as target genes for establishing nucleic acid-based diagnostic assays (Zhang et al., 2016). Monoclonal antibodies (mAbs) are laboratory-produced molecules that target specific viral antigens on the surface of pathogen. They are valuable and indispensable tools for investigating the antigenic properties of the M protein (Dong et al., 2016). Moreover, the establishment of a monoclonal antibody-producing hybridoma cell line specific for the CoV. M protein has laid a solid foundation for CoV identification and the development of related diagnostic methods. When developing molecular diagnostic methods (such as RT-PCR) for CoVs, researchers typically design specific primers and probes targeting the hydrophilic N-terminal coding region of the M gene (Xin et al., 2024). Compared with the high variability of the S protein, using the highly conserved M protein as a diagnostic antigen can theoretically improve the stability and accuracy of detection. However, this approach remains in the early stage of research and is far from clinical application.

### 4.3 M protein is a promising candidate for vaccine design

The M protein is not only the most abundant viral structural protein but also exhibits high conservation of its amino acid sequence. Specific antibodies recognize its extracellular domain, and this high degree of conservative makes it a promising candidate for a broad-spectrum vaccine. Vaccines based on the M protein therefore hold significant advantages. First, its high degree of conservation may enable cross-protective immunity against diverse CoVs, potentially even across species barriers. Second, in CoV, membrane fusion is mediated by the S protein, whereas the M protein does not participate in this process (Wang Q. et al., 2020). Vaccines developed based on the S protein carry certain risks (Amanat and Krammer, 2020), whereas the distinction between the S and M proteins suggests that vaccines targeting the M protein may be associated with a lower risk of vaccine-associated antibody-dependent enhancement (ADE). Additionally, the M protein can simultaneously stimulate antibody production and a robust T-cell immune response in the host, which may contribute to a more balanced and potentially long-lasting immunity. The antibody response to the extracellular domain of M protein likely confers broad protective benefits against diverse SARS-CoV-2 variants in the population, exerting this effect through cross-protection both *in vitro* and *in vivo* (Tang et al., 2024). This finding highlights the value of the M protein as a promising and important antigenic target for designing effective CoV vaccines. The majority of recent vaccines are based on the S protein as the immunogen to stimulate protective immunity against SARS-CoV-2 in humans (Corbett et al., 2020; Liu et al., 2021). In individuals who have recovered from COVID-19, the proportion of CD8 + T cells specific for the M protein exceeds that specific for the S protein (Peng et al., 2020). This finding highlights the need to include M protein epitopes in future vaccine designs (Mattoo and Myoung, 2022). Recently, researchers have identified a cluster of polyfunctional, CD4-restricted T-cell epitopes in a highly conserved region of the SARS-CoV-2 M protein. The epitopes that elicit polyfunctional T-cell responses are promising targets for developing effective vaccines and T-cell-based therapies, as they are designed to induce broad, protective immunity (Grifoni et al., 2020; Keller et al., 2020; Khadri et al., 2024). Currently, in clinical practice, there is no vaccine that uses the M protein as the primary immunogen. However, in models such as that of PDCoV, multi-antigen vaccines based on VLPs (containing S, M, and E proteins) have demonstrated superior efficacy (Liu et al., 2023a). The VLPs induce high levels of serum-specific IgG and virus-neutralizing (VN) antibodies in mice. Furthermore, incorporating both the N and M proteins into the vaccine can elicit a stronger cellular immune response, promoting CD8 + T-cell expansion and IFN-γ production (Yu et al., 2024). Compared with vaccines that express only the S protein, the multi-antigen vaccine can elicit a stronger and broader immune response in the host.

### 4.4 M protein is the key proteins for studying the mechanisms of CoVs infection

The M protein is indispensable for the process of virus assembly and release. Studying the structure, function, and interactions of the M protein with other viral or host cell proteins provides insights into the fundamental biological processes of CoVs, including infection pathogenesis, the viral replication cycle, and the molecular mechanisms underlying essential steps in virus assembly. Furthermore, the high-level expression of the M protein in infected cells makes it an ideal model system for studying glycosylation and ER-to-Golgi vesicular trafficking (Fung and Liu, 2018).

## 5 Discussion

The M protein is the most abundant structural protein in viral particles and plays a crucial role in viral assembly, protein-protein interactions, morphogenesis, host interactions, and immune regulation. In recent years, with the global spread of SARS-CoV-2, a series of important advances have been made in research on the M protein. The CoV M protein has a three-transmembrane-domain structure, and its dimerization is essential for viral envelope formation. By interacting with other viral structural proteins and ensuring their capture and coordinated incorporation at the budding site, the M protein orchestrates virion assembly (Tusnády et al., 2015). The M protein acts in synergy with the E protein to facilitate the formation of the viral envelope. It interacts with the N protein to promote the packaging of the RNP into viral particles. The M protein can inhibit the type-I interferon signaling pathway, evade immune recognition via glycosylation, and interact with the host vesicular transport system to promote viral release. The M protein also regulates apoptosis.

In structure studies of the M protein, although numerous studies have been conducted, many questions remain need to be addressed. The structural study has confirmed that the TM domain of the M protein is highly hydrophobic and lacks a detectable ion conduction pathway. However, its potential role in ion conduction cannot be excluded. It is unclear whether its oligomers can function as ion channels, and further research is needed to confirm this. Moreover, the M protein is highly conserved among CoVs, but its glycosylation patterns and specific interactions with other viral proteins exhibit variability. Do these discrepancies influence its function? How do distinct glycan moieties attached to the M protein affect its functional properties? Viral replication is highest in strains expressing an N-glycosylated M protein; however, the precise role of M protein N-glycosylation in CoVs remains incompletely understood (Juckel et al., 2023). A precise assessment of how M protein glycosylation influences its function still requires the development of more specific and sensitive analytical methods. With the development of mass spectrometry technology, new protein enrichment and analysis techniques have greatly enhanced the sensitivity and accuracy of glycosylation modification site identification, which may help advance research on the aforementioned issues. Specifically, current research has proposed a new model suggesting that the M protein of MERS-CoV contains four α-helical, hydrophobic transmembrane domains. And both its N-terminal and C-terminal domains are oriented toward the extracellular side of the viral envelope (Alharbi and Alrefaei, 2021). At present, research confirming this inference is limited. If this hypothesis holds true, the additional (fourth) transmembrane domain may stabilize the binding of M protein to the viral envelope or the host cell membrane, thereby enhancing the overall structural stability of the viral envelope. Moreover, the additional transmembrane region and its associated ring-like structure may expose new binding surfaces that mediate specific interactions with other M proteins, auxiliary viral factors, or host membrane proteins, which may affect viral assembly or immune modulation.

The M protein holds significant importance and considerable potential value in immunological detection, the development of novel anti-CoV drugs, and vaccine research. In developing immunological targets for coronavirus detection, the M protein is typically used as an antigen in ELISA. In the *Escherichia coli* expression system, the M protein can be expressed at high levels, facilitating the preparation and standardization of ELISAs based on recombinant M protein (Elia et al., 2003). Furthermore, the availability of substantial quantities of recombinant M protein provides an opportunity to gain deeper insights into the biological functions of the M protein and its immunological role during CoV infection. In terms of research on drugs against CoVs, small molecules or polypeptide inhibitors can interfere with M-M protein interactions, thereby disrupting the formation of the viral envelope scaffold. By interfering with the interaction between the M and N proteins, this approach is expected to prevent recruitment of the viral genome to the assembly sites. When developing antiviral drugs, locking the M protein into a specific conformation to block virion assembly represents a key strategy (Laporte et al., 2025; Van Damme et al., 2025). The M protein is highly conserved, and drugs developed against it may have broad-spectrum activity against CoVs. In addition, the M protein holds significant potential as a supplementary antigen in vaccine development. Although the M protein exhibits a limited ability to induce neutralizing antibodies, it can elicit high-titer binding antibodies. Meanwhile, the M protein contains abundant and highly conserved T-cell epitopes that can strongly induce CD4 + and CD8 + T-cell responses. These responses are crucial for eliminating infected cells and establishing long-term immune memory. The emergence of the novel human SARS-CoV-2 virus has underscored the critical and urgent need for continuous monitoring of viral infections, the development of practical antivirals against CoVs, and the development of innovative and more effective vaccines (Chalupka et al., 2024; Polatoğlu et al., 2023; Zhu et al., 2022). At the same time, the structural similarities among the M proteins of different CoVs suggest that developing a common inhibitor targeting these CoVs is feasible. Research suggests that the mutation in the M protein may be induced by co-occurring mutations in the spike protein receptor-binding domain. This finding is of great significance for vaccine development and therapeutic strategies (Ye et al., 2024). Expression both the S and M proteins in a vaccine can trigger a stronger immune response than expressing only the S protein (Bellier et al., 2022). This stronger immune response is specifically manifested by vaccines containing both proteins, which induce high levels of serum IgA and mucosal IgA. A study has found that in the vaccine breakthrough infection (VBI) cohort, immunity against the wild-type SARS-CoV-2 and its S proteins declined after receiving the primary booster vaccine against COVID-19 (Paniskaki et al., 2022). This result indicates that the neutralizing efficacy of vaccines based solely on the S protein as an antigen is limited.

Currently, there are still many challenges in the research on M protein applications. For instance, the immunogenicity of the M protein is relatively weak, potent adjuvants or advanced delivery systems are needed; structural analysis—particularly of its transmembrane domain—remains technically challenging, hindering the rational design of targeted therapeutics; and clinical data on M protein-based vaccines is still insufficient, warranting further validation through well-designed trials. At present, research on the S protein of CoVs is more in-depth and extensive. As CoVs continue to mutate and new epidemics emerge, developing an M protein–based “second pillar” strategy represents a wise and prudent approach. This complementary strategy augments the current S protein-centric paradigm and is essential for constructing a robust, multi-layered defense system against CoVs.

This article reviews the structure, biological functions, and potential applications of the CoV M protein. Much remains unknown about the M protein and requires further study, such as the immunomodulatory function of distinct M protein mutants, the effect of the M protein on host cell metabolism, and its inhibitory effects on CoV infection. In addition to the major structural proteins, other accessory proteins are also present in the CoVs. Although these proteins are not always essential for viral replication, they may play certain roles in processes such as viral packaging and immune evasion. However, interactions between the M protein and these accessory proteins, as well as their precise functions, remain unclear and require rigorous experimental validation. Other critical questions related to the M protein are also worthy of exploration: Do M protein mutants elicit cross-reactive T-cell responses against heterologous CoVs? What is the therapeutic efficacy of M protein-targeted inhibitors *in vivo*? And how does M protein expression modulate antigen presentation or dendritic cell activation? Addressing these questions will highlight promising directions for future research. Furthermore, the M protein has demonstrated considerable value in laboratory studies and warrants further investigation into its potential clinical applications. In summary, this review synthesizes current knowledge on the CoV M protein, providing a foundational reference to enhance mechanistic understanding of its biological functions and to inform assessments of its potential as a therapeutic target and for vaccine development.

## References

[B1] Aebi M. (2013). N-linked protein glycosylation in the ER. *Biochim. Biophys. Acta* 1833 2430–2437. 10.1016/j.bbamcr.2013.04.001 23583305

[B2] Albalawi W. Thomas J. Mughal F. Kotsiri A. Roper K. Alshehri A. (2025). SARS-CoV-2 S, M, and E structural glycoproteins differentially modulate endoplasmic reticulum stress responses. *Int. J. Mol. Sci.* 26:1047. 10.3390/ijms26031047 39940816 PMC11816748

[B3] Alharbi S. Alrefaei A. (2021). Comparison of the SARS-CoV-2 (2019-nCoV) M protein with its counterparts of SARS-CoV and MERS-CoV species. *J. King Saud. Univ. Sci.* 33:101335. 10.1016/j.jksus.2020.101335 33432259 PMC7787911

[B4] Amanat F. Krammer F. (2020). SARS-CoV-2 vaccines: Status report. *Immunity* 52 583–589. 10.1016/j.immuni.2020.03.007 32259480 PMC7136867

[B5] Arndt A. Larson B. Hogue B. G. (2010). A conserved domain in the coronavirus membrane protein tail is important for virus assembly. *J. Virol.* 84 11418–11428. 10.1128/JVI.01131-10 20719948 PMC2953170

[B6] Bai Z. Cao Y. Liu W. Li J. (2021). The SARS-CoV-2 nucleocapsid protein and its role in viral structure, biological functions, and a potential target for drug or vaccine mitigation. *Viruses* 13:1115. 10.3390/v13061115 34200602 PMC8227405

[B7] Bellier B. Saura A. Luján L. Molina C. Luján H. Klatzmann D. A. (2022). Thermostable oral SARS-CoV-2 vaccine induces mucosal and protective immunity. *Front. Immunol.* 13:837443. 10.3389/fimmu.2022.837443 35281065 PMC8913903

[B8] Bhowmik D. Nandi R. Jagadeesan R. Kumar N. Prakash A. Kumar D. (2020). Identification of potential inhibitors against SARS-CoV-2 by targeting proteins responsible for envelope formation and virion assembly using docking based virtual screening, and pharmacokinetics approaches. *Infect. Genet. Evol.* 84:104451. 10.1016/j.meegid.2020.104451 32640381 PMC7335633

[B9] Boson B. Legros V. Zhou B. Siret E. Mathieu C. Cosset F. (2021). The SARS-CoV-2 envelope and membrane proteins modulate maturation and retention of the spike protein, allowing assembly of virus-like particles. *J. Biol. Chem.* 296:100111. 10.1074/jbc.RA120.016175 33229438 PMC7833635

[B10] Bracquemond D. Muriaux D. (2021). Betacoronavirus assembly: Clues and perspectives for elucidating SARS-CoV-2 particle formation and egress. *mBio* 12:e0237121. 10.1128/mBio.02371-21 34579570 PMC8546641

[B11] Brian D. Baric R. (2005). Coronavirus genome structure and replication. *Curr. Top. Microbiol. Immunol.* 287 1–30. 10.1007/3-540-26765-4_1 15609507 PMC7120446

[B12] Castaño N. Cordts S. Kurosu Jalil M. Zhang K. Koppaka S. Bick A. (2021). Fomite transmission, physicochemical origin of virus-surface interactions, and disinfection strategies for enveloped viruses with applications to SARS-CoV-2. *ACS Omega* 6 6509–6527. 10.1021/acsomega.0c06335 33748563 PMC7944398

[B13] Chalupka A. Richter L. Chakeri A. El-Khatib Z. Theiler-Schwetz V. Trummer C. (2024). Effectiveness of a fourth SARS-CoV-2 vaccine dose in previously infected individuals from Austria. *Eur. J. Clin. Invest.* 54:e14136. 10.1111/eci.14136 38032853 PMC11475503

[B14] Chan C. Ma C. Chan W. Chan H. (2007). The SARS-Coronavirus Membrane protein induces apoptosis through modulating the Akt survival pathway. *Arch. Biochem. Biophys.* 459 197–207. 10.1016/j.abb.2007.01.012 17306213 PMC7094499

[B15] Chang C. Hou M. Chang C. Hsiao C. Huang T. (2014). The SARS coronavirus nucleocapsid protein–forms and functions. *Antiviral Res.* 103 39–50. 10.1016/j.antiviral.2013.12.009 24418573 PMC7113676

[B16] Collins L. Elkholy T. Mubin S. Hill D. Williams R. Ezike K. (2021). Elucidation of SARS-Cov-2 budding mechanisms through molecular dynamics simulations of M and E protein complexes. *J. Phys. Chem. Lett.* 12 12249–12255. 10.1021/acs.jpclett.1c02955 34928612

[B17] Corbett K. Edwards D. Leist S. Abiona O. Boyoglu-Barnum S. Gillespie R. (2020). SARS-CoV-2 mRNA vaccine design enabled by prototype pathogen preparedness. *Nature* 586 567–571. 10.1038/s41586-020-2622-0 32756549 PMC7581537

[B18] Cubuk J. Alston J. Incicco J. Singh S. Stuchell-Brereton M. Ward M. (2021). The SARS-CoV-2 nucleocapsid protein is dynamic, disordered, and phase separates with RNA. *Nat. Commun.* 12 021–21953. 10.1038/s41467-021-21953-3 33782395 PMC8007728

[B19] de Haan C. de Wit M. Kuo L. Montalto-Morrison C. Haagmans B. Weiss S. (2003). The glycosylation status of the murine hepatitis coronavirus M protein affects the interferogenic capacity of the virus in vitro and its ability to replicate in the liver but not the brain. *Virology* 312 395–406. 10.1016/s0042-6822(03)00235-6 12919744 PMC7126936

[B20] de Haan C. Vennema H. Rottier P. (2000). Assembly of the coronavirus envelope: Homotypic interactions between the M proteins. *J. Virol.* 74 4967–4978. 10.1128/jvi.74.11.4967-4978.2000 10799570 PMC110848

[B21] Desmarets L. Danneels A. Burlaud-Gaillard J. Blanchard E. Dubuisson J. Belouzard S. (2023). The KxGxYR and DxE motifs in the C-tail of the Middle East respiratory syndrome coronavirus membrane protein are crucial for infectious virus assembly. *Cell. Mol. Life Sci.* 80:353. 10.1007/s00018-023-05008-y 37940699 PMC10632273

[B22] Dominguez S. Sims G. Wentworth D. Halpin R. Robinson C. Town C. (2012). Genomic analysis of 16 Colorado human NL63 coronaviruses identifies a new genotype, high sequence diversity in the N-terminal domain of the spike gene and evidence of recombination. *J. Gen. Virol.* 93 2387–2398. 10.1099/vir.0.044628-0 22837419 PMC4091283

[B23] Dong H. Zhang X. Shi H. Chen J. Shi D. Zhu Y. (2016). Characterization of an immunodominant epitope in the endodomain of the coronavirus membrane protein. *Viruses* 8:327. 10.3390/v8120327 27973413 PMC5192388

[B24] Elia G. Fiermonte G. Pratelli A. Martella V. Camero M. Cirone F. (2003). Recombinant M protein-based ELISA test for detection of antibodies to canine coronavirus. *J. Virol. Methods* 109 139–142. 10.1016/s0166-0934(03)00064-8 12711056 PMC7119567

[B25] Escors D. Camafeita E. Ortego J. Laude H. Enjuanes L. (2001a). Organization of two transmissible gastroenteritis coronavirus membrane protein topologies within the virion and core. *J. Virol.* 75 12228–12240. 10.1128/JVI.75.24.12228-12240.2001 11711614 PMC116120

[B26] Escors D. Ortego J. Laude H. Enjuanes L. (2001b). The membrane M protein carboxy terminus binds to transmissible gastroenteritis coronavirus core and contributes to core stability. *J. Virol.* 75 1312–1324. 10.1128/JVI.75.3.1312-1324.2001 11152504 PMC114037

[B27] Farsani S. Dijkman R. Jebbink M. Goossens H. Ieven M. Deijs M. (2012). The first complete genome sequences of clinical isolates of human coronavirus 229E. *Virus Genes* 45 433–439. 10.1007/s11262-012-0807-9 22926811 PMC7088690

[B28] Fu Y. Wang S. Zheng Z. Yi H. Li W. W. Xu Z. S. (2021). SARS-CoV-2 membrane glycoprotein M antagonizes the MAVS-mediated innate antiviral response. *Cell. Mol. Immunol.* 18 613–620. 10.1038/s41423-020-00571-x 33110251 PMC7588591

[B29] Fung T. Liu D. (2018). Post-translational modifications of coronavirus proteins: Roles and function. *Future Virol.* 13 405–430. 10.2217/fvl-2018-0008 32201497 PMC7080180

[B30] Fung T. Liu D. (2019). Human coronavirus: Host-pathogen interaction. *Annu. Rev. Microbiol.* 73 529–557. 10.1146/annurev-micro-020518-115759 31226023

[B31] Grifoni A. Weiskopf D. Ramirez S. Mateus J. Dan J. Moderbacher C. (2020). Targets of T *Cell* responses to SARS-CoV-2 coronavirus in humans with COVID-19 disease and unexposed individuals. *Cell* 181 1489–1501.e15. 10.1016/j.cell.2020.05.015 32473127 PMC7237901

[B32] Han Y. Zhou H. Liu C. Wang W. Qin Y. Chen M. (2024). SARS-CoV-2 N protein coordinates viral particle assembly through multiple domains. *J Virol.* 98:e0103624. 10.1128/jvi.01036-24 39412257 PMC11575404

[B33] He Y. Zhou Y. Siddiqui P. Niu J. Jiang S. (2005). Identification of immunodominant epitopes on the membrane protein of the severe acute respiratory syndrome-associated coronavirus. *J. Clin. Microbiol.* 43 3718–3726. 10.1128/JCM.43.8.3718-3726.2005 16081901 PMC1234014

[B34] Hsieh Y. Li H. Chen S. Lo S. (2008). Interactions between M protein and other structural proteins of severe, acute respiratory syndrome-associated coronavirus. *J. Biomed. Sci.* 15 707–717. 10.1007/s11373-008-9278-3 18792806 PMC7089546

[B35] Hui X. Zhang L. Cao L. Huang K. Zhao Y. Zhang Y. (2021). SARS-CoV-2 promote autophagy to suppress type I interferon response. *Signal. Transduct Target Ther.* 6:180. 10.1038/s41392-021-00574-8 33966045 PMC8105701

[B36] Hurst K. Koetzner C. Masters P. (2009). Identification of in vivo-interacting domains of the murine coronavirus nucleocapsid protein. *J. Virol.* 83 7221–7234. 10.1128/JVI.00440-09 19420077 PMC2704785

[B37] Ji Z. Dong H. Jiao R. Zhu X. Shi H. Chen J. (2023). The TGEV membrane protein interacts with HSC70 to direct virus internalization through clathrin-mediated endocytosis. *J. Virol.* 97:e0012823. 10.1128/jvi.00128-23 36975782 PMC10134871

[B38] Juckel D. Desmarets L. Danneels A. Rouillé Y. Dubuisson J. Belouzard S. (2023). MERS-CoV and SARS-CoV-2 membrane proteins are modified with polylactosamine chains. *J. Gen. Virol.* 104:1900. 10.1099/jgv.0.001900 37800895

[B39] Keller M. Harris K. Jensen-Wachspress M. Kankate V. Lang H. Lazarski C. (2020). SARS-CoV-2-specific T cells are rapidly expanded for therapeutic use and target conserved regions of the membrane protein. *Blood* 136 2905–2917. 10.1182/blood.2020008488 33331927 PMC7746091

[B40] Khadri L. Ziraksaz M. Barekzai A. Ghauri B. (2024). T cell responses to SARS-CoV-2. *Prog. Mol. Biol. Transl. Sci.* 202 183–217. 10.1016/bs.pmbts.2023.06.001 38237986

[B41] Kindler E. Thiel V. Weber F. (2016). Interaction of SARS and MERS coronaviruses with the antiviral interferon response. *Adv. Virus Res.* 96 219–243. 10.1016/bs.aivir.2016.08.006 27712625 PMC7112302

[B42] Ko C. Kang M. Lim G. Kim G. Yoon S. Park J. (2006). Molecular characterization of HE, M, and E genes of winter dysentery bovine coronavirus circulated in Korea during 2002-2003. *Virus Genes* 32 129–136. 10.1007/s11262-005-6867-3 16604443 PMC7089456

[B43] Kuo L. Masters P. (2013). Functional analysis of the murine coronavirus genomic RNA packaging signal. *J. Virol.* 87 5182–5192. 10.1128/JVI.00100-13 23449786 PMC3624306

[B44] Kuo L. Hurst-Hess K. Koetzner C. Masters P. (2016a). Analyses of coronavirus assembly interactions with interspecies membrane and nucleocapsid protein chimeras. *J. Virol.* 90 4357–4368. 10.1128/JVI.03212-15 26889024 PMC4836358

[B45] Kuo L. Koetzner C. Masters P. S. (2016b). A key role for the carboxy-terminal tail of the murine coronavirus nucleocapsid protein in coordination of genome packaging. *Virology* 494 100–107. 10.1016/j.virol.2016.04.009 27105451 PMC4884538

[B46] Kuo L. Koetzner C. Hurst K. Masters P. (2014). Recognition of the murine coronavirus genomic RNA packaging signal depends on the second RNA-binding domain of the nucleocapsid protein. *J. Virol.* 88 4451–4465. 10.1128/JVI.03866-13 24501403 PMC3993769

[B47] Lai C. Chan Z. Yang D. Lo W. Lai Y. Chang M. (2006). Accelerated induction of apoptosis in insect cells by baculovirus-expressed SARS-CoV membrane protein. *FEBS Lett*. 580 3829–3834. 10.1016/j.febslet.2006.06.003 16797548 PMC7094299

[B48] Laporte M. Jochmans D. Bardiot D. Desmarets L. Debski-Antoniak O. Mizzon G. (2025). A coronavirus assembly inhibitor that targets the viral membrane protein. *Nature* 640 514–523. 10.1038/s41586-025-08773-x 40140569 PMC11981944

[B49] Lau S. Woo P. Li K. Huang Y. Wang M. Lam C. (2007). Complete genome sequence of bat coronavirus HKU2 from Chinese horseshoe bats revealed a much smaller spike gene with a different evolutionary lineage from the rest of the genome. *Virology* 367 428–439. 10.1016/j.virol.2007.06.009 17617433 PMC7103351

[B50] Li G. Fan Y. Lai Y. Han T. Li Z. Zhou P. (2020). Coronavirus infections and immune responses. *J. Med. Virol.* 92 424–432. 10.1002/jmv.25685 31981224 PMC7166547

[B51] Li S. Zhu Z. Yang F. Cao W. Yang J. Ma C. (2021). Porcine epidemic diarrhea virus membrane protein interacted with IRF7 to inhibit type I IFN production during viral infection. *J. Immunol.* 206 2909–2923. 10.4049/jimmunol.2001186 34127522

[B52] Li Y. Li C. Zhao C. Wu J. Zhu Y. Wang F. (2024). Coronavirus M protein promotes mitophagy over virophagy by recruiting PDPK1 to phosphorylate SQSTM1 at T138. *Nat. Commun.* 15:8927. 10.1038/s41467-024-53100-z 39414765 PMC11484861

[B53] Liang J. Fang S. Yuan Q. Huang M. Chen R. Fung T. (2019). N-Linked glycosylation of the membrane protein ectodomain regulates infectious bronchitis virus-induced ER stress response, apoptosis and pathogenesis. *Virology* 531 48–56. 10.1016/j.virol.2019.02.017 30852271 PMC7112112

[B54] Lin C. Chan K. Ooi E. Chiou M. Hoang M. Hsueh P. (2021). Animal coronavirus diseases: Parallels with COVID-19 in humans. *Viruses* 13:1507. 10.3390/v13081507 34452372 PMC8402828

[B55] Lin P. Wang M. Wei Y. Kim T. Wei X. (2020). Coronavirus in human diseases: Mechanisms and advances in clinical treatment. *MedComm* 1 270–301. 10.1002/mco2.26 33173860 PMC7646666

[B56] Liu G. Carter B. Gifford D. (2021). Predicted cellular immunity population coverage gaps for SARS-CoV-2 subunit vaccines and their augmentation by compact peptide sets. *Cell. Syst.* 12 102–107.e4. 10.1016/j.cels.2020.11.010 33321075 PMC7691134

[B57] Liu J. Sun Y. Qi J. Chu F. Wu H. Gao F. (2010). The membrane protein of severe acute respiratory syndrome coronavirus acts as a dominant immunogen revealed by a clustering region of novel functionally and structurally defined cytotoxic T-lymphocyte epitopes. *J. Infect. Dis.* 202 1171–1180. 10.1086/656315 20831383 PMC7537489

[B58] Liu Y. Han X. Qiao Y. Wang T. Yao L. (2023a). Porcine deltacoronavirus-like particles produced by a single recombinant baculovirus elicit virus-specific immune responses in mice. *Viruses* 15:1095. 10.3390/v15051095 37243181 PMC10221120

[B59] Liu Y. Lu T. Li C. Wang X. Chen F. Yue L. (2023b). Comparative transcriptome analysis of SARS-CoV-2, SARS-CoV, MERS-CoV, and HCoV-229E identifying potential IFN/ISGs targets for inhibiting virus replication. *Front. Med.* 10:1267903. 10.3389/fmed.2023.1267903 38143441 PMC10739311

[B60] Lopez L. Riffle A. Pike S. Gardner D. Hogue B. (2008). Importance of conserved cysteine residues in the coronavirus envelope protein. *J. Virol.* 82 3000–3010. 10.1128/JVI.01914-07 18184703 PMC2258990

[B61] Lu S. Xie X. Zhao L. Wang B. Zhu J. Yang T. (2021). The immunodominant and neutralization linear epitopes for SARS-CoV-2. *Cell. Rep.* 34:108666. 10.1016/j.celrep.2020.108666 33503420 PMC7837128

[B62] Lui P. Wong L. Fung C. Siu K. Yeung M. Yuen K. (2016). Middle East respiratory syndrome coronavirus M protein suppresses type I interferon expression through the inhibition of TBK1-dependent phosphorylation of IRF3. *Emerg. Microbes Infect.* 5:e39. 10.1038/emi.2016.33 27094905 PMC4855074

[B63] Mahtarin R. Islam S. Islam M. Ullah M. Ali M. Halim M. (2022). Structure and dynamics of membrane protein in SARS-CoV-2. *J. Biomol. Struct. Dyn*. 40 4725–4738. 10.1080/07391102.2020.1861983 33353499 PMC7784837

[B64] Malik Y. (2020). Properties of coronavirus and SARS-CoV-2. *Malays J. Pathol.* 42 3–11. 32342926

[B65] Marques-Pereira C. Pires M. Gouveia R. Pereira N. Caniceiro A. Rosário-Ferreira N. (2022). SARS-CoV-2 membrane protein: From genomic data to structural new insights. *Int. J. Mol. Sci.* 23:2986. 10.3390/ijms23062986 35328409 PMC8948900

[B66] Marra M. Jones S. Astell C. Holt R. Brooks-Wilson A. Butterfield Y. (2003). The genome sequence of the SARS-associated coronavirus. *Science* 300 1399–1404. 10.1126/science.1085953 12730501

[B67] Masters P. (2019). Coronavirus genomic RNA packaging. *Virology* 537 198–207. 10.1016/j.virol.2019.08.031 31505321 PMC7112113

[B68] Mattoo S. Myoung J. T. (2022). cell responses to SARS-CoV-2 in humans and animals. *J. Microbiol.* 60 276–289. 10.1007/s12275-022-1624-z 35157219 PMC8852923

[B69] Miranda C. Silva V. Igrejas G. Poeta P. (2021). Genomic evolution of the human and animal coronavirus diseases. *Mol. Biol. Rep.* 48 6645–6653. 10.1007/s11033-021-06632-2 34383242 PMC8358252

[B70] Mizutani T. Fukushi S. Iizuka D. Inanami O. Kuwabara M. Takashima H. (2006a). Inhibition of cell proliferation by SARS-CoV infection in Vero E6 cells. *FEMS Immunol. Med. Microbiol.* 46 236–243. 10.1111/j.1574-695X.2005.00028.x 16487305 PMC7110397

[B71] Mizutani T. Fukushi S. Saijo M. Kurane I. Morikawa S. (2006b). Regulation of p90RSK phosphorylation by SARS-CoV infection in Vero E6 cells. *FEBS Lett.* 580 1417–1424. 10.1016/j.febslet.2006.01.066 16458888 PMC7094696

[B72] Monje-Galvan V. Voth G. (2021). Molecular interactions of the M and E integral membrane proteins of SARS-CoV-2. *Faraday Discuss.* 232 49–67. 10.1039/d1fd00031d 34543372 PMC8712422

[B73] Mounir S. Talbot P. (1992). Sequence analysis of the membrane protein gene of human coronavirus OC43 and evidence for O-glycosylation. *J. Gen. Virol.* 73 2731–2736. 10.1099/0022-1317-73-10-2731 1402806

[B74] Mukherjee S. Bhattacharyya D. Bhunia A. (2020). Host-membrane interacting interface of the SARS coronavirus envelope protein: Immense functional potential of C-terminal domain. *Biophys. Chem.* 266:106452. 10.1016/j.bpc.2020.106452 32818817 PMC7418743

[B75] Müller M. van der Hoek L. Voss D. Bader O. Lehmann D. Schulz A. (2010). Human coronavirus NL63 open reading frame 3 encodes a virion-incorporated N-glycosylated membrane protein. *Virol. J.* 7:6. 10.1186/1743-422X-7-6 20078868 PMC2819038

[B76] Nal B. Chan C. Kien F. Siu L. Tse J. Chu K. (2005). Differential maturation and subcellular localization of severe acute respiratory syndrome coronavirus surface proteins S. M and E. *J. Gen. Virol.* 86 1423–1434. 10.1099/vir.0.80671-0 15831954

[B77] Narayanan K. Makino S. (2001). Cooperation of an RNA packaging signal and a viral envelope protein in coronavirus RNA packaging. *J. Virol.* 75 9059–9067. 10.1128/JVI.75.19.9059-9067.2001 11533169 PMC114474

[B78] Narayanan K. Chen C. Maeda J. Makino S. (2003). Nucleocapsid-independent specific viral RNA packaging via viral envelope protein and viral RNA signal. *J. Virol.* 77 2922–2927. 10.1128/jvi.77.5.2922-2927.2003 12584316 PMC149775

[B79] Narayanan K. Maeda A. Maeda J. Makino S. (2000). Characterization of the coronavirus M protein and nucleocapsid interaction in infected cells. *J. Virol*. 74 8127–8134. 10.1128/jvi.74.17.8127-8134.2000 10933723 PMC112346

[B80] Naskalska A. Dabrowska A. Szczepanski A. Milewska A. Jasik K. Pyrc K. (2019). Membrane protein of human coronavirus NL63 is responsible for interaction with the adhesion receptor. *J. Virol.* 93:e00355-19. 10.1128/JVI.00355-19 31315999 PMC6744225

[B81] Neuman B. Kiss G. Kunding A. Bhella D. Baksh M. Connelly S. (2011). A structural analysis of M protein in coronavirus assembly and morphology. *J. Struct. Biol.* 174 11–22. 10.1016/j.jsb.2010.11.021 21130884 PMC4486061

[B82] Ng O. Chia A. Tan A. Jadi R. Leong H. Bertoletti A. (2016). Memory T cell responses targeting the SARS coronavirus persist up to 11 years post-infection. *Vaccine* 34 2008–2014. 10.1016/j.vaccine.2016.02.063 26954467 PMC7115611

[B83] Pang H. Liu Y. Han X. Xu Y. Jiang F. Wu D. (2004). Protective humoral responses to severe acute respiratory syndrome-associated coronavirus: Implications for the design of an effective protein-based vaccine. *J. Gen. Virol.* 85 3109–3113. 10.1099/vir.0.80111-0 15448374

[B84] Paniskaki K. Anft M. Meister T. Marheinecke C. Pfaender S. Skrzypczyk S. (2022). Immune response in moderate to critical breakthrough COVID-19 infection after mRNA vaccination. *Front. Immunol.* 13:816220. 10.3389/fimmu.2022.816220 35145522 PMC8821964

[B85] Park J. Ryu J. Park J. Hong E. Shin H. (2021). Heat shock protein 70 could enhance porcine epidemic diarrhoea virus replication by interacting with membrane proteins. *Vet. Res.* 52:138. 10.1186/s13567-021-01006-9 34717778 PMC8557036

[B86] Park S. Kim H. Song D. An D. Park B. (2012). Complete genome sequences of a Korean virulent porcine epidemic diarrhea virus and its attenuated counterpart. *J. Virol.* 86 557–512. 10.1128/JVI.00557-12 22532530 PMC3347302

[B87] Park S. Kim H. Song D. Moon H. Park B. (2011). Molecular characterization and phylogenetic analysis of porcine epidemic diarrhea virus (PEDV) field isolates in Korea. *Arch. Virol.* 156 577–585. 10.1007/s00705-010-0892-9 21210162 PMC7086862

[B88] Peele K. Kumar V. Parate S. Srirama K. Lee K. Venkateswarulu T. (2021). Insilico drug repurposing using FDA approved drugs against Membrane protein of SARS-CoV-2. *J. Pharm. Sci.* 110 2346–2354. 10.1016/j.xphs.2021.03.004 33684397 PMC7934671

[B89] Peng Y. Mentzer A. Liu G. Yao X. Yin Z. Dong D. (2020). Broad and strong memory CD4+ and CD8+ T cells induced by SARS-CoV-2 in UK convalescent individuals following COVID-19. *Nat. Immunol.* 21 1336–1345. 10.1038/s41590-020-0782-6 32887977 PMC7611020

[B90] Perrier A. Bonnin A. Desmarets L. Danneels A. Goffard A. Rouillé Y. (2019). The C-terminal domain of the MERS coronavirus M protein contains a trans-Golgi network localization signal. *J. Biol. Chem.* 294 14406–14421. 10.1074/jbc.RA119.008964 31399512 PMC6768645

[B91] Polatoğlu I. Oncu-Oner T. Dalman I. Ozdogan S. (2023). COVID-19 in early 2023: Structure, replication mechanism, variants of SARS-CoV-2, diagnostic tests, and vaccine & drug development studies. *MedComm* 4:e228. 10.1002/mco2.228 37041762 PMC10082934

[B92] Polyiam K. Ruengjitchatchawalya M. Mekvichitsaeng P. Kaeoket K. Hoonsuwan T. Joiphaeng P. (2022). Immunodominant and neutralizing linear B-cell epitopes spanning the spike and membrane proteins of porcine epidemic diarrhea virus. *Front. Immunol.* 12:785293. 10.3389/fimmu.2021.785293 35126354 PMC8807655

[B93] Ren X. Suo S. Jang Y. (2011). Development of a porcine epidemic diarrhea virus M protein-based ELISA for virus detection. *Biotechnol. Lett.* 33 215–220. 10.1007/s10529-010-0420-8 20882317 PMC7088053

[B94] Rodríguez-Enríquez A. Herrera-Camacho I. Millán-Pérez-Peña L. Reyes-Leyva J. Santos-López G. Rivera-Benítez J. (2022). Predicted 3D model of the M protein of porcine epidemic diarrhea virus and analysis of its immunogenic potential. *PLoS One* 17:e0263582. 10.1371/journal.pone.0263582 35139120 PMC8827446

[B95] Satarker S. Nampoothiri M. (2020). Structural proteins in severe acute respiratory syndrome Coronavirus-2. *Arch. Med. Res.* 51 482–491. 10.1016/j.arcmed.2020.05.012 32493627 PMC7247499

[B96] Schoeman D. Fielding B. (2019). Coronavirus envelope protein: Current knowledge. *Virol. J*. 16 019–1182. 10.1186/s12985-019-1182-0 31133031 PMC6537279

[B97] Schwarze M. Volke D. Rojas Echeverri J. Schick R. Lakowa N. Grünewald T. (2024). Influence of mutations and N-Glycosylation sites in the receptor-binding domain (RBD) and the membrane protein of SARS-CoV-2 variants of concern on antibody binding in ELISA. *Biology* 13:207. 10.3390/biology13040207 38666819 PMC11047955

[B98] Shrimal S. Cherepanova N. Gilmore R. (2015). Cotranslational and posttranslocational N-glycosylation of proteins in the endoplasmic reticulum. *Semin. Cell. Dev. Biol.* 41 71–78. 10.1016/j.semcdb.2014.11.005 25460543 PMC4442082

[B99] Siu K. Kok K. Ng M. Poon V. Yuen K. Zheng B. (2009). Severe acute respiratory syndrome coronavirus M protein inhibits type I interferon production by impeding the formation of TRAF3.TANK.TBK1/IKKepsilon complex. *J. Biol. Chem.* 284 16202–16209. 10.1074/jbc.M109.008227 19380580 PMC2713514

[B100] Song D. Park B. (2012). Porcine epidemic diarrhoea virus: A comprehensive review of molecular epidemiology, diagnosis, and vaccines. *Virus Genes*. 44 167–175. 10.1007/s11262-012-0713-1 22270324 PMC7089188

[B101] Steiner S. Kratzel A. Barut G. Lang R. Aguiar Moreira E. Thomann L. (2024). SARS-CoV-2 biology and host interactions. *Nat. Rev. Microbiol.* 22 206–225. 10.1038/s41579-023-01003-z 38225365

[B102] Sun G. Cui Q. Garcia G. Wang C. Zhang M. Arumugaswami V. (2021). Comparative transcriptomic analysis of SARS-CoV-2 infected cell model systems reveals differential innate immune responses. *Sci. Rep.* 11:17146. 10.1038/s41598-021-96462-w 34433867 PMC8387424

[B103] Swann H. Sharma A. Preece B. Peterson A. Eldredge C. Belnap D. (2020). Minimal system for assembly of SARS-CoV-2 virus like particles. *Sci. Rep.* 10:21877. 10.1038/s41598-020-78656-w 33318562 PMC7736577

[B104] Tang F. Quan Y. Xin Z. Wrammert J. Ma M. Lv H. (2011). Lack of peripheral memory B cell responses in recovered patients with severe acute respiratory syndrome: A six-year follow-up study. *J. Immunol.* 186 7264–7268. 10.4049/jimmunol.0903490 21576510

[B105] Tang Y. Tang K. Hu Y. Ye Z. Luo W. Luo C. (2024). M protein ectodomain-specific immunity restrains SARS-CoV-2 variants replication. *Front. Immunol.* 15:1450114. 10.3389/fimmu.2024.1450114 39416782 PMC11480003

[B106] Thomas S. (2020). The structure of the membrane protein of SARS-CoV-2 resembles the sugar transporter SemiSWEET. *Pathog. Immun*. 5 342–363. 10.20411/pai.v5i1.377 33154981 PMC7608487

[B107] Tseng Y. Chang C. Wang S. Huang K. Wang C. (2013). Identifying SARS-CoV membrane protein amino acid residues linked to virus-like particle assembly. *PLoS One* 8:e64013. 10.1371/journal.pone.0064013 23700447 PMC3659117

[B108] Tseng Y. Wang S. Huang K. Lee A. Chiang C. Wang C. (2010). Self-assembly of severe acute respiratory syndrome coronavirus membrane protein. *J. Biol. Chem.* 285 12862–12872. 10.1074/jbc.M109.030270 20154085 PMC2857088

[B109] Tsoi H. Li L. Chen Z. Lau K. Tsui S. Chan H. (2014). The SARS-coronavirus membrane protein induces apoptosis via interfering with PDK1-PKB/Akt signalling. *Biochem. J.* 464 439–447. 10.1042/BJ20131461 25271362

[B110] Tusnády G. Dobson L. Tompa P. (2015). Disordered regions in transmembrane proteins. *Biochim. Biophys. Acta* 1848 2839–2848. 10.1016/j.bbamem.2015.08.002 26275590

[B111] Ujike M. Taguchi F. (2015). Incorporation of spike and membrane glycoproteins into coronavirus virions. *Viruses* 7 1700–1725. 10.3390/v7041700 25855243 PMC4411675

[B112] Van Damme E. Abeywickrema P. Yin Y. Xie J. Jacobs S. Mann M. (2025). A small-molecule SARS-CoV-2 inhibitor targeting the membrane protein. *Nature* 640 506–513. 10.1038/s41586-025-08651-6 40140563 PMC11981937

[B113] Voss D. Kern A. Traggiai E. Eickmann M. Stadler K. Lanzavecchia A. (2006). Characterization of severe acute respiratory syndrome coronavirus membrane protein. *FEBS Lett.* 580 968–973. 10.1016/j.febslet.2006.01.026 16442106 PMC7094741

[B114] Voss D. Pfefferle S. Drosten C. Stevermann L. Traggiai E. Lanzavecchia A. (2009). Studies on membrane topology, N-glycosylation and functionality of SARS-CoV membrane protein. *Virol. J.* 6:79. 10.1186/1743-422X-6-79 19534833 PMC2705359

[B115] Wang F. Han H. Wang C. Wang J. Peng Y. Chen Y. (2024). SARS-CoV-2 membrane protein induces neurodegeneration via affecting Golgi-mitochondria interaction. *Transl. Neurodegener.* 13:68. 10.1186/s40035-024-00458-1 39726060 PMC11674522

[B116] Wang Q. Wei J. He J. Ming S. Li X. Huang X. (2024). HSP70 contributes to pathogenesis of fulminant hepatitis induced by coronavirus. *Int. Immunopharmacol.* 141:112963. 10.1016/j.intimp.2024.112963 39159560

[B117] Wang Q. Zhang Y. Wu L. Niu S. Song C. Zhang Z. (2020). Structural and functional basis of SARS-CoV-2 entry by using human ACE2. *Cell* 181 894–904.e9. 10.1016/j.cell.2020.03.045 32275855 PMC7144619

[B118] Wang R. Yu R. Chen B. Si F. Wang J. Xie C. (2020). Identification of host cell proteins that interact with the M protein of porcine epidemic diarrhea virus. *Vet. Microbiol.* 246:108729. 10.1016/j.vetmic.2020.108729 32605758 PMC7241372

[B119] Wang Y. Liu L. (2016). The membrane protein of severe acute respiratory syndrome coronavirus functions as a novel cytosolic pathogen-associated molecular pattern to promote beta interferon induction via a toll-like-receptor-related TRAF3-independent mechanism. *mBio* 7:e01872-15. 10.1128/mBio.01872-15 26861016 PMC4752600

[B120] Wang Y. Grunewald M. Perlman S. (2020). Coronaviruses: An updated overview of their replication and pathogenesis. *Methods Mol. Biol.* 2203 900–902. 10.1007/978-1-0716-0900-2_1 32833200 PMC7682345

[B121] Woo P. Lau S. Lam C. Lau C. Tsang A. Lau J. (2012). Discovery of seven novel Mammalian and avian coronaviruses in the genus deltacoronavirus supports bat coronaviruses as the gene source of alphacoronavirus and betacoronavirus and avian coronaviruses as the gene source of gammacoronavirus and deltacoronavirus. *J. Virol.* 86 3995–4008. 10.1128/JVI.06540-11 22278237 PMC3302495

[B122] Woo P. Wang M. Lau S. Xu H. Poon R. Guo R. (2007). Comparative analysis of twelve genomes of three novel group 2c and group 2d coronaviruses reveals unique group and subgroup features. *J. Virol.* 81 1574–1585. 10.1128/JVI.02182-06 17121802 PMC1797546

[B123] Wu H. Li C. Sun X. Cheng Y. Chen Z. (2023). Identification of a monoclonal antibody against porcine deltacoronavirus membrane protein. *Int. J. Mol. Sci.* 24:13934. 10.3390/ijms241813934 37762237 PMC10530725

[B124] Xin Z. Li S. Lu X. Liu L. Gao Y. Hu F. (2024). Development and clinical application of a molecular assay for four common porcine enteroviruses. *Vet. Sci.* 11:305. 10.3390/vetsci11070305 39057989 PMC11281614

[B125] Xu R. Shi M. Li J. Song P. Li N. (2020). Construction of SARS-CoV-2 virus-like particles by mammalian expression system. *Front. Bioeng. Biotechnol.* 8:862. 10.3389/fbioe.2020.00862 32850726 PMC7409377

[B126] Xu X. Zhang H. Zhang Q. Dong J. Huang Y. Tong D. (2015). Porcine epidemic diarrhea virus M protein blocks cell cycle progression at S-phase and its subcellular localization in the porcine intestinal epithelial cells. *Acta Virol.* 59 265–275. 10.4149/av_2015_03_265 26435150

[B127] Yamada Y. Yabe M. Ohtsuki T. Taguchi F. (2000). Unique N-linked glycosylation of murine coronavirus MHV-2 membrane protein at the conserved O-linked glycosylation site. *Virus Res.* 66 149–154. 10.1016/s0168-1702(99)00134-3 10725548 PMC7125849

[B128] Yang Y. Wu Y. Meng X. Wang Z. Younis M. Liu Y. (2022). SARS-CoV-2 membrane protein causes the mitochondrial apoptosis and pulmonary edema via targeting BOK. *Cell. Death Differ.* 29 1395–1408. 10.1038/s41418-022-00928-x 35022571 PMC8752586

[B129] Yang Y. Zhang L. Geng H. Deng Y. Huang B. Guo Y. (2013). The structural and accessory proteins M, ORF 4a, ORF 4b, and ORF 5 of Middle East respiratory syndrome coronavirus (MERS-CoV) are potent interferon antagonists. *Protein Cell.* 4 951–961. 10.1007/s13238-013-3096-8 24318862 PMC4875403

[B130] Ye Q. Wang H. Xu F. Zhang S. Zhang S. Yang Z. (2024). Co-Mutations and possible variation tendency of the spike RBD and membrane protein in SARS-CoV-2 by machine learning. *Int. J. Mol. Sci.* 25:4662. 10.3390/ijms25094662 38731879 PMC11083383

[B131] Yegnaswamy S. Selva Kumar C. Aldaais E. (2025). Conformational dynamics of the membrane protein of MERS-CoV in comparison with SARS-CoV-2 in ERGIC complex. *J. Biomol. Struct. Dyn.* 10.1080/07391102.2024.2437529 Online ahead of print. 39755960

[B132] Yu R. Zhang L. Zhou P. Zhang Z. Liu X. Wang Y. (2024). Evaluation of the immunoprotective effects of porcine deltacoronavirus subunit vaccines. *Virology* 590:109955. 10.1016/j.virol.2023.109955 38070302

[B133] Yuan Z. Ding B. (2024). Coronavirus hijacks STX18-ATG14 axis-regulated lipophagy to evade an anti-viral effect. *Autophagy* 20 1895–1896. 10.1080/15548627.2024.2330039 38477940 PMC11262221

[B134] Yuan Z. Cai K. Li J. Chen R. Zhang F. Tan X. (2024). ATG14 targets lipid droplets and acts as an autophagic receptor for syntaxin18-regulated lipid droplet turnover. *Nat. Commun.* 15:631. 10.1038/s41467-024-44978-w 38245527 PMC10799895

[B135] Yuan Z. Hu B. Xiao H. Tan X. Li Y. Tang K. (2021). The E3 ubiquitin ligase RNF5 facilitates SARS-CoV-2 membrane protein-mediated virion release. *mBio* 13:e0316821. 10.1128/mbio.03168-21 35100873 PMC8805027

[B136] Zambalde ÉP. Pavan I. C. B. Mancini M. C. S. Severino M. B. Scudero O. B. Morelli A. P. (2022). Characterization of the interaction between SARS-CoV-2 membrane protein (M) and proliferating cell nuclear antigen (PCNA) as a potential therapeutic target. *Front. Cell. Infect. Microbiol.* 12:849017. 10.3389/fcimb.2022.849017 35677658 PMC9168989

[B137] Zandi M. Soltani S. (2022). Severe acute respiratory syndrome coronavirus-2 and its structural proteins. *J. Cell. Physiol*. 237:9. 10.1002/jcp.30546 34352115 PMC8426883

[B138] Zhang C. Min Y. Xue H. Zhang H. Liu K. Tian Y. (2025). Host protein ARF1 is a proviral factor for SARS-CoV-2 and a candidate broad-spectrum therapeutic target. *Nat. Commun.* 16 025–61431. 10.1038/s41467-025-61431-8 40634337 PMC12241596

[B139] Zhang J. Ejikemeuwa A. Gerzanich V. Nasr M. Tang Q. Simard J. (2022). Understanding the role of SARS-CoV-2 ORF3a in viral pathogenesis and COVID-19. *Front. Microbiol.* 13:854567. 10.3389/fmicb.2022.854567 35356515 PMC8959714

[B140] Zhang J. Tsai Y. Lee P. Chen Q. Zhang Y. Chiang C. (2016). Evaluation of two singleplex reverse transcription-Insulated isothermal PCR tests and a duplex real-time RT-PCR test for the detection of porcine epidemic diarrhea virus and porcine deltacoronavirus. *J. Virol. Methods* 234 34–42. 10.1016/j.jviromet.2016.03.016 27060624 PMC7113669

[B141] Zhang Q. Chen Z. Huang C. Sun J. Xue M. Feng T. (2021). Severe acute respiratory syndrome Coronavirus 2 (SARS-CoV-2) membrane (M) and spike (S) proteins antagonize host type I interferon response. *Front. Cell. Infect. Microbiol.* 11:766922. 10.3389/fcimb.2021.766922 34950606 PMC8688923

[B142] Zhang Z. Nomura N. Muramoto Y. Ekimoto T. Uemura T. Liu K. (2022). Structure of SARS-CoV-2 membrane protein essential for virus assembly. *Nat. Commun.* 13:4399. 10.1038/s41467-022-32019-3 35931673 PMC9355944

[B143] Zhao H. Syed A. Khalid M. Nguyen A. Ciling A. Wu D. (2024). Assembly of SARS-CoV-2 nucleocapsid protein with nucleic acid. *Nucleic Acids Res.* 52 6647–6661. 10.1093/nar/gkae256 38587193 PMC11194069

[B144] Zhao Y. Liu X. Cheng J. Wu Y. Zhang G. (2014). Molecular characterization of an infectious bronchitis virus strain isolated from northern China in 2012. *Arch. Virol.* 159 3457–3461. 10.1007/s00705-014-2213-1 25168045 PMC7086801

[B145] Zheng Y. Zhuang M. Han L. Zhang J. Nan M. Zhan P. (2020). Severe acute respiratory syndrome coronavirus 2 (SARS-CoV-2) membrane (M) protein inhibits type I and III interferon production by targeting RIG-I/MDA-5 signaling. *Signal. Transduct Target Ther.* 5:299. 10.1038/s41392-020-00438-7 33372174 PMC7768267

[B146] Zhou S. Lv P. Li M. Chen Z. Xin H. Reilly S. (2023). SARS-CoV-2 E protein: Pathogenesis and potential therapeutic development. *Biomed. Pharmacother.* 159:114242. 10.1016/j.biopha.2023.114242 36652729 PMC9832061

[B147] Zhou Y. Wu W. Xie L. Wang D. Ke Q. Hou Z. (2017). Cellular RNA helicase DDX1 is involved in transmissible gastroenteritis virus nsp14-induced interferon-beta production. *Front. Immunol.* 8:940. 10.3389/fimmu.2017.00940 28848548 PMC5552718

[B148] Zhu Z. Zhang S. Wang P. Chen X. Bi J. Cheng L. (2022). A comprehensive review of the analysis and integration of omics data for SARS-CoV-2 and COVID-19. *Brief. Bioinform.* 23:bbab446. 10.1093/bib/bbab446 34718395 PMC8574485

[B149] Zhuang T. Yang P. Wang M. Liu S. Wang W. Sun B. (2025). Coronavirus M protein impairs cilium during early infection by enhancing the AurA-HDAC6 axis. *PLoS Pathog.* 21:e1013515. 10.1371/journal.ppat.1013515 40938964 PMC12445521

[B150] Zou H. Zarlenga D. Sestak K. Suo S. Ren X. (2013). Transmissible gastroenteritis virus: Identification of M protein-binding peptide ligands with antiviral and diagnostic potential. *Antiviral Res.* 99 383–390. 10.1016/j.antiviral.2013.06.015 23830854 PMC7114267

